# The Virtual-Environment-Foraging Task enables rapid training and single-trial metrics of rule acquisition and reversal in head-fixed mice

**DOI:** 10.1038/s41598-019-41250-w

**Published:** 2019-03-18

**Authors:** Martha N. Havenith, Peter M. Zijderveld, Sabrina van Heukelum, Shaghayegh Abghari, Paul Tiesinga, Jeffrey C. Glennon

**Affiliations:** Donders Institute for Brain, Cognition and Behaviour, Kapittelweg, 29 6525EN Nijmegen The Netherlands

## Abstract

Behavioural flexibility is an essential survival skill, yet our understanding of its neuronal substrates is still limited. While mouse research offers unique tools to dissect the neuronal circuits involved, the measurement of flexible behaviour in mice often suffers from long training times, poor experimental control, and temporally imprecise binary (hit/miss) performance readouts. Here we present a virtual-environment task for mice that tackles these limitations. It offers fast training of vision-based rule reversals (~100 trials per reversal) with full stimulus control and continuous behavioural readouts. By generating multiple non-binary performance metrics per trial, it provides single-trial estimates not only of response accuracy and speed, but also of underlying processes like choice certainty and alertness (discussed in detail in a companion paper). Based on these metrics, we show that mice can predict new task rules long before they are able to execute them, and that this delay varies across animals. We also provide and validate single-trial estimates of whether an error was committed with or without awareness of the task rule. By tracking in unprecedented detail the cognitive dynamics underlying flexible behaviour, this task enables new investigations into the neuronal interactions that shape behavioural flexibility moment by moment.

## Introduction

In an ever-changing environment, the ability to flexibly associate sensory input with different behavioural responses is essential to survival. For instance, a food source (say, a piece of cheese) may be worth approaching in most circumstances, but not in the context of imminent danger (e.g. a mouse trap or a nearby cat). Versatile cognitive mapping allows an animal to base different behavioural decisions on identical – or similar – sensory input.

The cortical circuits that allow us to accomplish such flexible rerouting between stimulus and response are thought to involve a wide network of areas^1^, including prefrontal^2–5^ and orbitofrontal cortex^6–8^, mediodorsal thalamus^4^, (basolateral) amygdala^5,6,8,9^ and striatum^10–12^. Yet in trying to further decipher these circuits and their dynamic contribution to flexible behaviour, neuroscience currently faces an obstacle: Species like primates and humans, who easily learn and execute flexible rule changes e.g. ^13–15^, do not lend themselves to the invasive and/or genetics-based techniques that are currently most powerful in dissecting the workings of neuronal networks. On the other hand, rodents, and particularly mice, offer a staggering arsenal of tools for the targeted recording and manipulation of neuronal population activity^16–28^. As a result, rodent research is becoming an increasingly valuable component in mapping the neuronal interactions that generate complex cognitive processes like rule learning. The issue here is that the behavioural paradigms assessing rule learning and rule reversal in mice often run into several limitations, most prominently long training and imprecise characterization of behaviour^29–31^.

Currently available tasks that measure flexible rule learning in mice generally employ one of two main task structures: First, direct reversal paradigms simply convert the original reward rule into its opposite (and back)^5,7,8,32^. Second, set shifting paradigms ask animals to alternate between two sets of rules that are not directly opposed to each other^33–38^. A typical example would be a set of visual stimuli that can be discriminated either based on colour or orientation. The two competing rules can be realised in the same sensory modality^33,36^ or in two different sensory modalities^35,37,38^.

Both reversal and set switching tasks have been employed in rodents to generate important fundamental insights into the neuronal circuits enabling flexible response selection. For instance, they have revealed a functional dissociation between orbitofrontal and medial prefrontal cortex: While interactions between orbitofrontal cortex and basolateral amygdala are necessary in order to acquire rule reversals (but not attentional set shifting)^6,8,9^, a network comprising medial prefrontal cortex and thalamus seems to drive attentional set shifting (but not rule reversal)^2,4,39,40^. Recent findings have particularly highlighted the role of interneurons in orchestrating the prefrontal and orbitofrontal responses that drive flexible behavior^2,4,32,40,41^. However, while we have a good understanding of which neuronal circuits are crucially involved in different aspects of behavioural flexibility, the question how these circuits interact moment-by-moment in order to generate a particular (flexible or inflexible) behavioural decision is far from solved.

One of the reasons for this gap in our knowledge is that current tests of behavioural flexibility in mice generally seem to face a steep trade-off between experimental control and behavioural ease: On the one hand, paradigms that prioritize learning efficiency and ecological validity capitalize on innate mouse behaviour (e.g. digging for food), and target the dominant sensory systems of mice (arguably olfaction and somatosensation)^12,32,35,38^. Such naturalistic approaches achieve great training efficiency: Animals can reverse rules and accomplish set shifts within a dozen trials or less^2,3,35,38^ – a speed that is comparable to humans and primates^13–15,34,42^. However, this comes at a cost: Animals only complete small numbers of trials per session, limiting subsequent analyses; head fixation is impossible, complicating neuronal recording and manipulation; and neither sensory stimuli nor behavioural responses can be precisely controlled or recorded, often requiring manual interventions by the experimenter. Thus, while naturalistic task implementations evoke efficient rule learning, they make it difficult to accurately characterize the learning process and relate it to ongoing neuronal activity.

On the other hand, paradigms that use human/primate tasks as a template^33,36,39,43^ offer precise control and quantification of sensory stimuli and behavioural responses. By employing visual stimuli, they also make it easy to relate findings to the large body of knowledge gained with the use of visual tasks in other species. The drawback here is training speed: A single rule reversal requires 15–40 training sessions^44,45^, while an attentional set shift takes 10–15 sessions (in addition to 10–20 sessions of pre-shift training)^33^. This not only poses a logistical problem (e.g. for experiments in young animals), but also a conceptual issue: The fact that rule changes are learned on a completely different time scale than in other species (weeks as opposed to minutes) suggests that we may not be investigating the same cognitive process at all. Thus, tasks that allow for sufficient control to quantify behaviour in detail do not seem to evoke the flexible learning we have been studying in other species. While some task implementations manage to navigate between these extremes, featuring semi-naturalistic setups and intermediate training durations^40,46^, the underlying trade-off between experimental control and learning efficiency has so far remained.

Most importantly, all paradigms discussed so far face the common issue that they generate behavioural read-outs with limited power to characterize underlying learning processes in a nuanced way or on a trial-by-trial basis. Response quantification in individual trials is usually confined to a binary hit/miss classification. Occasionally, response latencies are also estimated, based either on manual records, or on an automatically recorded time-to-target. With these metrics alone, it is difficult to decipher the cognitive processes that lead to a specific behavioural choice in any given trial. For instance, an incorrect trial could be attributed to failed reversal learning – or to perceptual difficulty, frustration, low motivation, motor errors (e.g. accidental licking) and so on. Similarly, a long time-to-target may indicate that an animal made its target choice late in the trial and/or that it was running slowly. The fact that metrics of response timing are either unavailable or only loosely related to the actual moment of response choice makes it difficult to link behaviour to ongoing neuronal dynamics in a precise way.

The issue of incomplete response quantification continues on the scale of the overall learning process: Based on the single-trial metrics discussed above, currently available paradigms generally determine the moment of rule acquisition using a pre-defined criterion point (e.g. 85% correct performance). Error trials are then often classified as ‘perseverative’ (errors committed before the criterion point, thought to indicate incomplete rule acquisition) or ‘regressive’ (errors committed after the criterion point, thought to indicate an intermittent regression to the old rule)^2,4,5,32^. But can we truly assume that the moment when an animal achieves, say, 85% correct performance is directly related to the moment when the animal becomes explicitly aware of a new task rule, and that all (or most) previous errors were due to failed rule learning, while all (or most) subsequent ones were produced by a momentary resurfacing of the old rule (rather than any of the competing behavioural factors like stimulus difficulty, attentional lapses, low motivation etc.)? This seems unlikely, meaning that the performance metrics we currently extract from rule-change paradigms are not robust or detailed enough to fully support the interpretations we want to derive from them. As such, we are left with little scope for analysing an animal’s learning process in depth, or to relate it directly to ongoing neuronal dynamics.

Here we present a paradigm for rule learning and rule reversal in mice that aims to address these obstacles. Our paradigm is based on a virtual-environment^46–48^ foraging (VEF) task in which animals approach a visual target (while disregarding a distractor) in order to obtain reward. By relying on innate foraging behavior, the VEF task shortens training times significantly compared to other vision-based paradigms of reversal learning. At the same time, head fixation makes it easy to relate behaviour directly to neuronal population recording and manipulation. Finally, continuous tracking of multiple single-trial performance metrics allows us to characterize rule learning trial-by-trial, relying on data-driven analyses that reduce the need for pre-defined criterion points. As a result, we are able to dissociate the moment of rule learning from that of rule execution, demonstrating that animals often predict the correct target up to 150 trials before they succeed in consistently approaching it.

## Results

**Figure 1:**
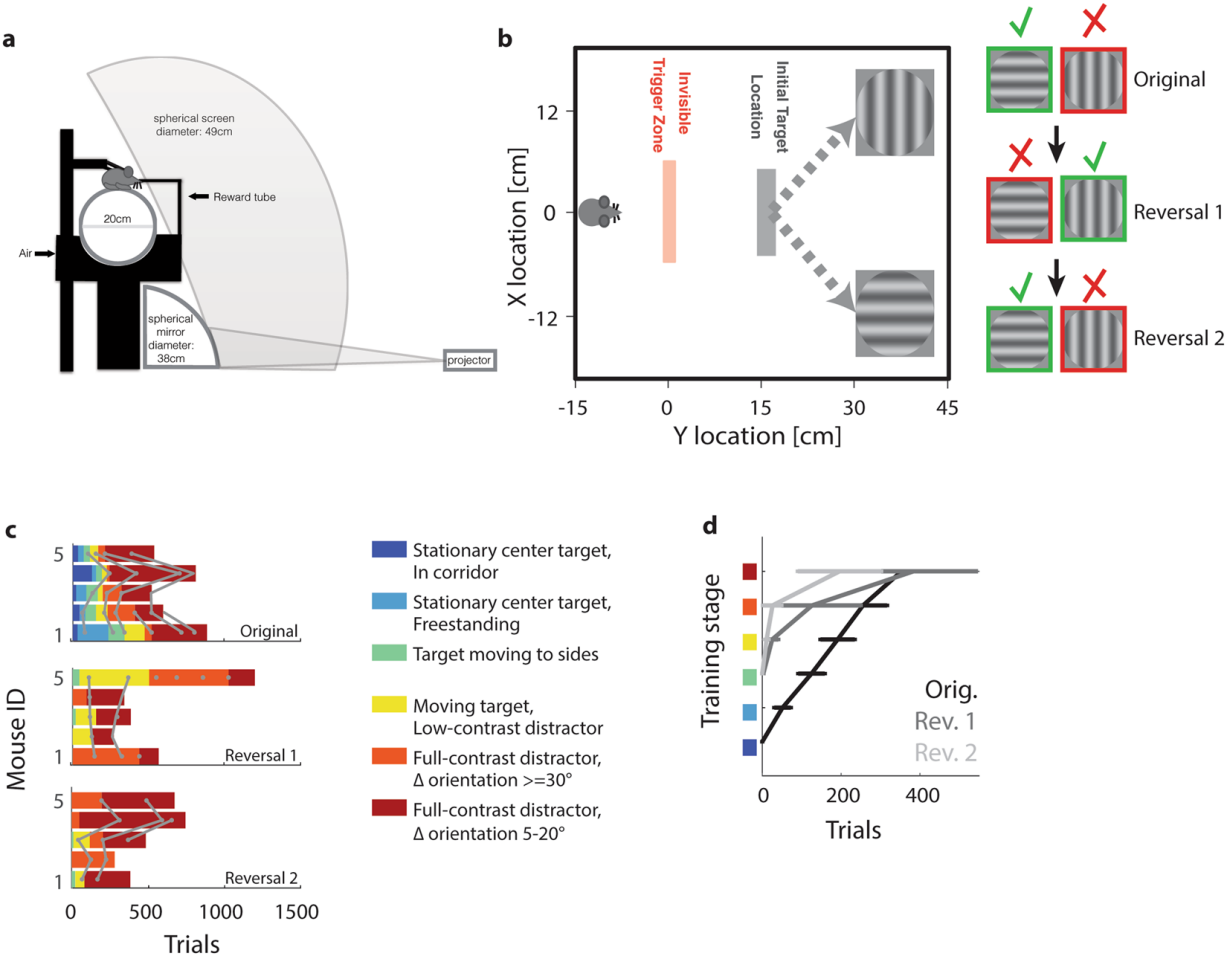
Task structure and training. (**a**) Schematic of experimental setup. (**b**) Schematic of the VEF task, with virtual coordinates converted to distance traversed on the treadmill. When an animal crosses the invisible trigger zone, a grey target originally located at the centre moves to the side and displays a circular sinusoidal grating, while a distractor displaying a differently oriented grating moves to the opposite side. Right-hand inset: Reward contingencies for original task and two rule reversals. Note that animals are presented with stimulus pairs of varying (and increasingly difficult) orientation differences, beginning with 90° differences (horizontal versus vertical, see inset) and advancing to 5° differences (42.5° versus 47.5°). (**c**) Top panel: Training progress of five animals in the original task, as a function of the number of trials completed. Training is colour-coded in six stages (see inset legend). After training, animals typically completed 2–5 sessions of the final task (i.e. training stage 6) for statistical evaluation of performance. Here, the first two sessions of training stage 6 are shown for each animal. Grey lines: Transitions between training sessions. Bottom panels: same for first and second rule reversal, respectively. (**d**) Average training progression of five animals as a function of the number of completed trials, shown for the original task (black), first (dark grey) and second (light grey) reversal. Error bars: SEM. Training stages are color-coded as in panel c.

The paradigm presented here relies on an immersive virtual environment setup, adapted from the one described by^47^, and detailed in^49^. In short, food-deprived animals were head-fixed on a floating-ball treadmill surrounded by a spherical projection surface (Fig. 1a). A grey target initially appears in front of the mouse. As the animal approaches the target, it crosses an invisible trigger threshold. This causes the target to move to the left, centre or right, and display a circular sinusoidal grating. Centre trials function as instruction trials (see Methods), simply requiring the animal to run straight ahead for reward. They are therefore not analysed further. For targets that move to the side, a distractor simultaneously moves in the opposite direction and displays a differently oriented grating (Fig. 1b). In the easiest trials, target gratings were horizontal, while distractors were vertical (90° orientation difference). The hardest trials contained a 42.5° target and a 47.5° distractor (5° orientation difference). Animals received a cue tone and soymilk when they touched the target. If they touched the distractor, they received a punishment tone, subsequently entered a time-out corridor and then restarted the same trial. Like centre-target trials, repeat trials were implemented for instruction, and not analysed further (for details, see Methods and^49^, as well as Supplementary Movie S1).

### Task training and reversal learning

One of the advantages of the VEF task for studying rule acquisition and reversal is that it was optimized extensively for learning speed. To do so, we applied a seven-step training scheme, which was developed according to seven principles of task design for mice (see Supplementary Note and Supplementary Fig. S1 for a full description and training examples). In short, to optimize learning speed and task performance, we minimized physical discomfort, reduced stress e.g. by extended handling and by converting aversive punishments into trade-offs, and used response schemes that capitalize on innate behaviours (approaching a target to ‘forage for’ a reward; for related training approaches focused on visual discrimination, see^46,50^). For instance, extensive handling pre-training significantly increased training speed, as did the individual optimization of parameters like treadmill position (Supplementary Fig. S1). This led animals to grasp the association between targets and reward extremely quickly: Animals began anticipatory licking on approach to the target within less than 15 minutes of initial training (Supplementary Fig. S1).

To test the efficiency of the VEF task for rule and reversal learning, six animals that succeeded in trials with an orientation difference (**Δ**Ori) of 5° were subsequently trained in two rule reversals, exchanging the stimuli associated with reward and punishment. Note that out of these six animals, one did not learn the reversed rule, because it simply stopped initiating trials and licking for reward (data not shown). Animals were initially trained in seven consecutive steps (see Methods and Supplementary Movie S1), and for each rule reversal, the last four steps of training were repeated with reversed reward contingencies. Figure 1c shows the number of trials required to progress to each training step during initial task learning as well as during two rule reversals. In both task acquisition and subsequent rule reversals, most animals learned to perform coarse orientation discrimination within less than 300 trials, or four training sessions, and performed fine-grained orientation discrimination within less than 500 trials or 5–6 sessions. What’s more, learning seemed to become progressively faster throughout the two rule reversals: While during initial training mice required 166 to 477 trials (median: 223) or 2 to 8 sessions (median: 3) to reach training stage 5 (coarse orientation discrimination), they reached the same performance in 10 to 500 trials (median: 15) or 1 to 3 sessions (median:1) for the first rule reversal and in 11 to 153 trials (median: 76) or 1 to 2 sessions (median: 2) for the second reversal (Fig. 1d; for an example of training progress across original task and two reversals, see Supplementary Fig. S2). While this is slower than the training for some non-vision-based tasks^2,8,32,35^, it is drastically faster than comparable visual paradigms of rule reversal^44,45^.

### Primary and secondary performance metrics

**Figure 2:**
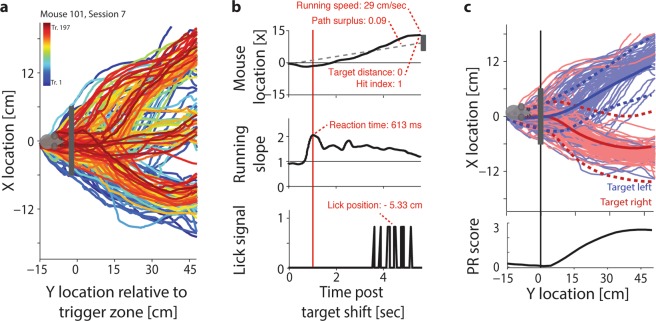
Computation of primary performance metrics. (**a**) Running trajectories from one training session in the original task, comprising 197 trials. Trials occurring later in the session are drawn in warmer colours. Mouse icon: Reset position at the trial start. Grey rectangle: Trigger zone for target shifts. Targets were located at either -12, 0 or 12 cm in the lateral (X) dimension. Note that the animal approaches the targets increasingly directly throughout the session. (**b**) Illustration of the extraction of six primary metrics from each running trajectory. Top panel: One running trajectory from the session shown in panel a, with lateral (X) position plotted as a function of time rather than Y location. Grey rectangle: Position of the target, used to compute the target distance. Dashed line: ‘Ideal’ direct path connecting the animal’s decision point to the target centre, used to compute the path surplus. Centre panel: Change in running slope for the same trial, showing a clear deflection shortly after the target shift. Red line: Time of largest deflection in running slope, used to measure the reaction time. Bottom panel: Trace of licking for the same trial, with each lick denoted as 1, used to compute the average lick position. (**c**) Illustration of the extraction of the seventh primary metric – the PR score - from running trajectories. Top panel: Same as in panel 1a, but showing only trials for left (blue) and right (red) target positions. Dark lines: Mean path per target location. Dark dashed lines: Mean path ± 1 Std.Dev. Bottom panel: PR score measuring the reliability of running trajectories to each target location, computed for each Y location as the normalized difference between all X locations for left and right targets. Note that we computed PR scores both globally over entire sessions, as shown here, and time-resolved, using a 15-trial sliding window (see Methods).

By working with head-fixed animals in a virtual environment, the VEF paradigm enables us to record a continuous stream of well-controlled readouts of running and licking (Fig. 2a). Such continuous behavioural tracking serves as the basis for a range of non-binary single-trial metrics capturing the timing and accuracy of task responses (Fig. 2b,c). These in turn can be used to infer second-tier metrics estimating higher-level cognitive processes such as attention and rule learning. The computation of both primary and secondary metrics is presented in detail in^49^. In short, we used the continuous recordings of running and licking to determine seven primary metrics of response accuracy and speed per trial (Fig. 2b,c): (1) The hit index, defined as 1 for correct trials, 0 for undecided trials, and −1 for trials when the animal touched the distractor. (2) The target distance, i.e. the animal’s lateral distance from the target at trial offset. The target distance was normalized by the virtual task space, so that hit trials have a target distance of 0, trials in which animals ended up approximately one target location removed from the target (e.g. close to the left target position when the actual target was located in the centre) have a target distance of 1, and so on. (3) The path reliability (PR) score quantifies the reproducibility of running paths towards the same target using the effect size Cohen’s D^51^. The PR score decreases in the presence of error trials, and increases with correct trials, but also with the reproducibility of successful running trajectories (Fig. 2c). (4) The path surplus quantifies the efficiency with which animals approach their chosen target. It is defined as the length of the actual running trajectory divided by the ‘ideal’ path (if the animal had directly approached its target following the point of target choice). This metric particularly increases when animals ‘change their mind’ – running first towards one target, then towards the other. (5) The reaction time, measured by determining for each trial the moment of the most abrupt change in running direction (see Supplementary Fig. S3 for a detailed illustration). (6) The lick position, i.e. the average Y position at which animals licked for reward within the vicinity of the target, computed such that negative numbers denote anticipatory licking while positive numbers denote licking after reward dispensation. (7) The average running speed between target shift and trial offset. Details on all primary metrics can be found in Methods and^49^. For an example of how primary performance metrics evolve throughout task training, please see Supplementary Fig. S4.

**Figure 3:**
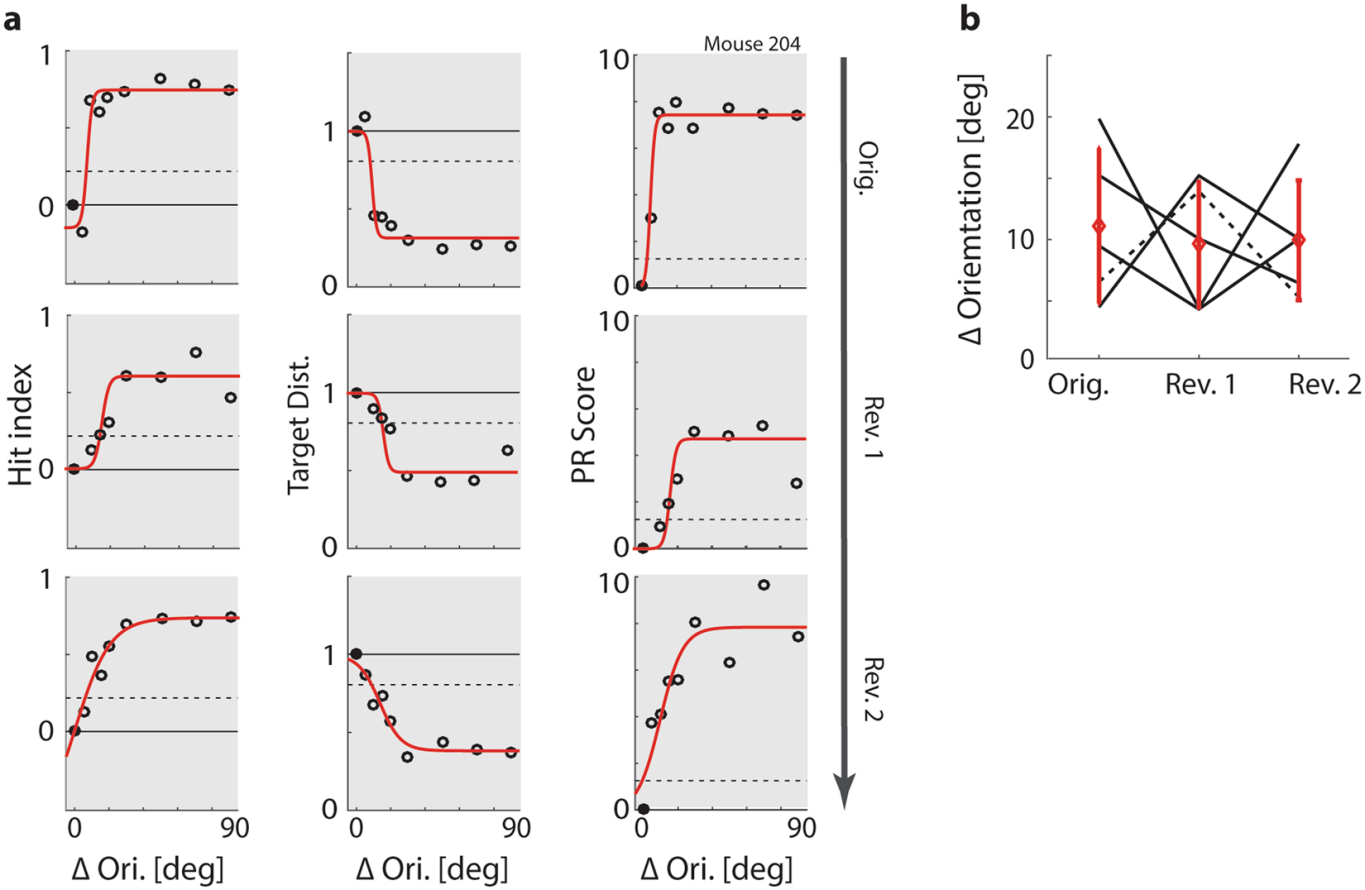
Animals can perform exact orientation discrimination. (**a**) Top left: Average hit index as a function of ΔOri for one animal in the original task. Circles: Average performance per ΔOri. Red line: Fitted sigmoid function. Black line: Chance level. Black dashed line: Criterion value for significant orientation discrimination (α = 0.05, see Methods). Top centre and top right: Same for target distance and PR score, respectively. Middle and bottom row: Same for first and second reversal, respectively. (**b**) Visual thresholds for five animals across original task and two rule reversals, estimated using a conjunction of hit index, target distance and PR score. Black lines: Animals. Dashed line: Animal shown in a. Red diamonds: Mean visual thresholds. Red lines: Mean ± Std.Dev. Correlations are shown above the panel (n = 5; all p > 0.05).

From these primary metrics of task performance, we computed six secondary metrics that quantify specific cognitive processes, including spontaneous fluctuations of alertness throughout the task, two complementary metrics of sustained and cued attention, and a metric of overall cognitive load, which quantifies performance irrespective of whether animals prioritized response accuracy or speed. These metrics are presented in detail in^49^. For the purposes of quantifying rule and reversal learning, three secondary metrics are particularly relevant: (1) The visual threshold of orientation discrimination, which was determined by taking into account three different psychometric curves: those of the hit index, target distance and PR score (shown in Fig. 3). To estimate individual visual thresholds, we defined critical values of discrimination for hit index, target distance and PR score based on a bootstrapping procedure (see Methods). The overall discrimination threshold was then computed by averaging the ΔOri at which the three critical values were reached^49^. (2) A classification of spontaneous ‘up’ versus ‘down’ states of alertness, derived from the bimodal distribution of local (15-trial sliding average) PR scores over time (used in Fig. 7). (3) The error prediction (EP) index, which estimates to what extent animals can predict whether or not their response will be rewarded. The EP index measures the difference in reaction time, path surplus, running speed and licking position in correct versus incorrect trials (for a detailed illustration of the computation steps involved, see Supplementary Fig. S5). Animals that have fully acquired the task show a later target choice, slower running, a less direct path towards the target and later licking in incorrect trials, suggesting that their decision making may be more hesitant. Such uncertainty can be seen to indicate that an animal has internalized the task rule and is therefore able to predict that a response is unlikely to result in reward (for a comparison of alternative interpretations, see Discussion). The EP index is particularly important in the context of studying reversal learning, because it allows us to quantify rule acquisition independently of rule execution. The implications of this dissociation are presented in detail in the following sections.

Extracting these single-trial metrics opens up several novel opportunities compared to alternative tasks. First, behavioural confounds are either controlled for or explicitly measured to a larger extent (see^52^). For instance, responses in this paradigm were entirely free of false positives, because changing running direction on the treadmill was sufficiently energy-consuming that animals never changed direction spontaneously in the absence of target stimuli^49^. Second, by tracking behaviour throughout the trial rather than focusing on its ultimate outcome (e.g. a screen touch), we are able to better estimate the moment of decision making within the trial – which can in turn be used to more precisely examine neuronal correlates of target choice. Finally, quantifying not only trial success but also reward anticipation and choice certainty allows us to track the dynamics of task learning in a more nuanced way.

### Visual discrimination remains stable across rule reversals

To test whether rule reversals impacted the visual performance of animals, we generated psychometric curves of orientation discrimination, and used them to compute the discrimination threshold after each rule reversal. Figure 3a shows psychometric curves across two rule reversals for one example animal (for average tuning curves of all primary metrics across original and reversed task, see Supplementary Fig. S4). Based on these psychometric curves of hit index, target distance and PR score, we estimated an overall threshold of orientation discrimination for each animal and each rule reversal (see^49^ for details). As Fig. 3b shows, animals generally reached extremely accurate discrimination performance: All animals consistently achieved discrimination thresholds below 20°, and 10 out of 15 thresholds (five animals across original task and two reversals) even remained below 10°. These estimates indicate significantly better visual acuity than previous measurements of orientation discrimination in mice^53–55^, demonstrating that although mouse vision is generally coarser than that of e.g. cats or primates^53,56–58^, in an adaptive, naturalistic task mice can nevertheless identify even minute orientation differences. Most importantly, visual discrimination did not appear to deteriorate across rule reversals. This suggests that the measured discrimination thresholds largely reflect genuine visual limitations rather than e.g. confusion or low motivation due to rule changes.

### Rule acquisition and error prediction

Subjectively, anyone who has ever played a game of Tetris or tried their luck at a tongue twister knows that understanding the rules of a game is not the same as executing them flawlessly. In human psychophysics, the gap between task acquisition and execution can be estimated for instance by asking subjects how certain they are of a particular choice^59–63^. If subjects understand the task in principle, but make errors e.g. due to stimulus difficulty, they will likely report lower choice certainty in error trials. For obvious reasons, a similar device has so far proven difficult to implement for mice (but see^64^ for an implementation in rats). As a result, it has been virtually impossible to dissociate errors arising from a suboptimal grasp of the task from perception-based errors, or indeed from deliberate rule breaking. Clearly, this is particularly crucial when studying the neuronal mechanisms underlying rule learning and rule reversal.

**Figure 4:**
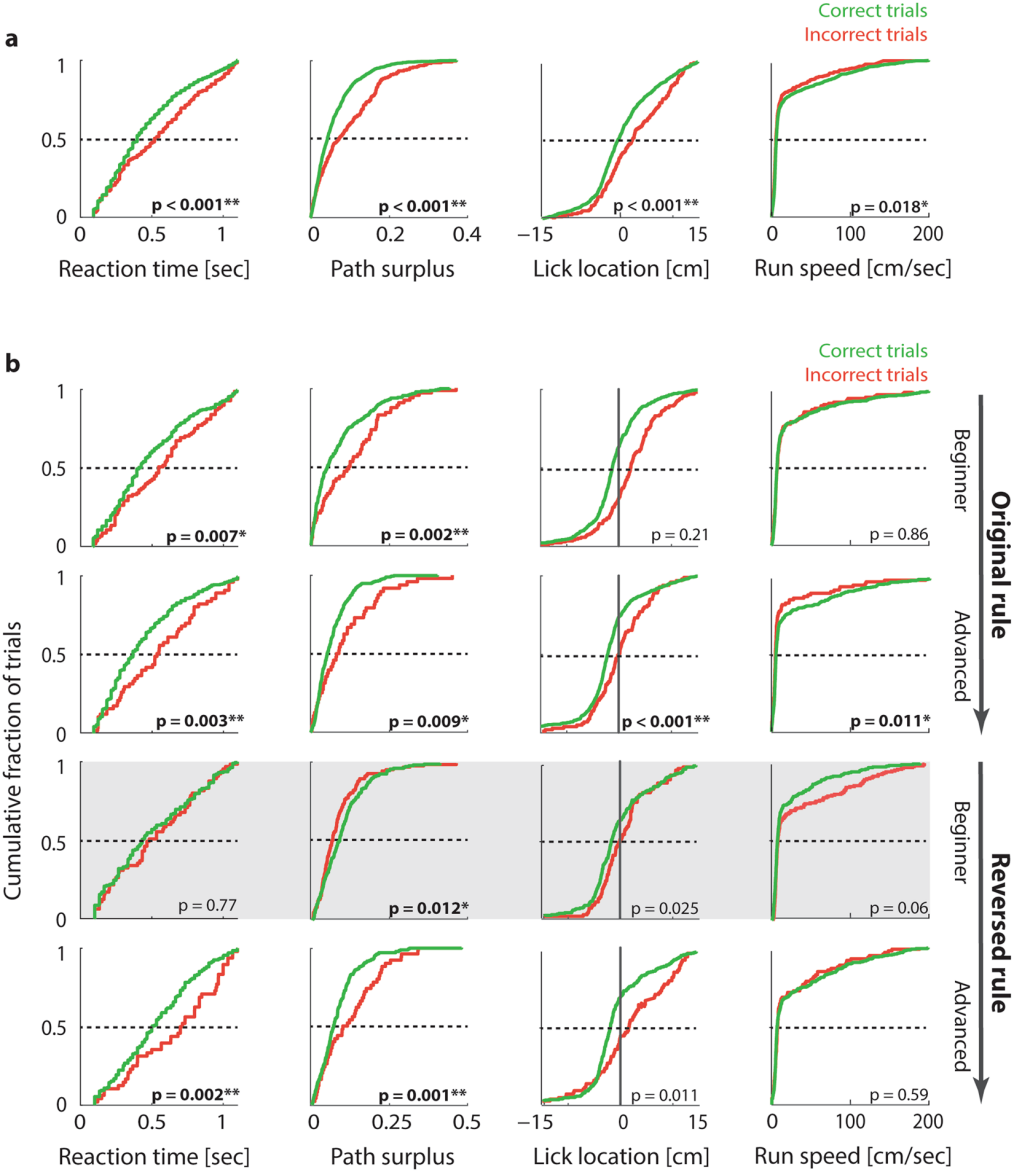
After rule acquisition mice are able to predict errors. (**a**) Differences in reaction time, path surplus, running speed and lick location for correct versus incorrect trials, pooled across five animals in the original task. Left panel: Cumulative distribution function (CDF) of reaction times, pooled across animals for all trials of the original task. Green: Correct trials. Red: Incorrect trials. Insets: P-values resulting from a Kolmogorov-Smirnov test of the distribution differences between correct and incorrect trials (*p < 0.05; **p < 0.01 after correction for multiple comparisons; see Methods). (**b**) Differences in reaction time, path surplus, running speed and lick location for correct versus incorrect trials, at different stages of rule acquisition. Top row: Cumulative distribution function (CDF) of reaction times (left), path surplus (centre left), lick positions (centre right) and running speed (right), for the first 30% of trials of ΔOri = 30° or easier, beginning at training stage 5, pooled across all animals. Green: Correct trials. Red: Incorrect trials. Insets: p-values resulting from a Kolmogorov-Smirnov test of the distribution differences between correct and incorrect trials (*p < 0.05; **p < 0.01 after correction for multiple comparisons; see Methods). Second row: Same for the last 30% of trials of ΔOri = 30° or easier in the original task. Third row: Same for the first 30% of trials of ΔOri = 30° or easier after the first rule reversal. Grey shade in background highlights that this is the time point when distribution differences are transiently diminished (reaction time, path surplus and licking position) and/or reversed (path surplus and running speed). Bottom row: Same for the final 30% of trials of ΔOri = 30° or easier after the first rule reversal.

In order to arrive at an estimate of task acquisition that could be obtained independently of rule execution, we first explored whether animals showed behavioural differences in correct versus incorrect trials. To this end we compared the primary performance metrics introduced above for correct and incorrect trials. Out of the six metrics that can in principle be related to trial success, two (target distance and PR score) were discarded because they are mathematically dependent on the hit index, and would therefore show spurious differences for correct and incorrect trials. Four performance metrics remain that are mathematically independent of the hit index: Reaction time, path surplus, running speed and lick position. Figure 4a shows the distribution of these four metrics for correct and incorrect trials across all animals throughout the original task (see Supplementary Fig. S6 for the same distributions in an individual animal). All four metrics indicate marked behavioural differences between correct and incorrect trials: In incorrect trials, animals appeared to react later, run more slowly, approach the target less directly and lick for reward later (Kolmogorov-Smirnov tests; df = 1275 based on 1277 trials in 5 animals; Reaction times: K-S statistic = 0.15; Path surplus: K-S = 0.22; Lick location: K-S = 0.17; Running speed: K-S = 0.08).

One interpretation consistent with these observations is that in incorrect trials, animals were less certain of their choice (leading to slower and more hesitant decision making), and predicted that they might not receive reward (leading to decreased anticipatory licking). This is exactly what one would expect to see if animals had indeed acquired the task rule and were using it to predict likely trial outcomes. In that case, animals might still make mistakes e.g. due to visual difficulty, but would in such trials be less certain of their choice, leading to hesitant behaviour.

However, other interpretations consistent with this behavioural pattern can also be envisioned. For instance, slow reaction times and late licking for reward might also occur when an animal paid little attention to the task or was otherwise limited in its processing capacity. This would at the same time make the animal more likely to choose the wrong target, leading to a correlation between hesitant reactions, late licking and incorrect target choice. A third potential explanation revolves around task familiarity: Error trials would be expected to occur more frequently at the start of training, which is also when running trajectories are still suboptimal, and animals may not yet anticipate reward consistently. The apparent behavioural difference between correct and incorrect trials would then simply arise from their relative distribution across training: All trials early in training would be equally slow and hesitant, while all trials late in training would be decisive and feature anticipatory licking. Since early trials are more commonly incorrect and late trials are more commonly correct, this would lead to an average difference in behaviour.

To gauge the relative likelihood of these alternative scenarios, we tracked how behavioural differences between correct and incorrect trials evolved over the course of task learning. This can serve as a benchmark test because the three potential explanations introduced above result in distinct predictions on how task learning should affect behaviour in correct and incorrect trials. Specifically, if the behavioural differences we observed are a by-product of task familiarity, behaviour at the beginning of training should be equally hesitant across correct and incorrect trials, and should then improve at an equal rate across training. Rule reversal should not affect this trend, since both the presented stimuli and the required responses would remain familiar, even if the association between them was switched. If on the other hand behavioural differences between correct and incorrect trials are due to fluctuating attention or processing capacity, there should be a largely stable difference throughout task learning, since low attention should affect performance approximately equally regardless of long or short training. Rule reversal should then only cause a short perturbation until animals began to grasp the reversed rule.

Finally, if the observed behavioural differences are a reflection of error prediction based on knowledge of the task rule, then they should be strongly affected by task learning: Animals that had not yet acquired the task should show similar behaviour for correct and incorrect choices, because they would presumably not anticipate different outcomes. Behavioural differences between correct and incorrect trials should then increase the more familiar animals became with the task, but should break down or even reverse after rule reversal. This was indeed the case: Fig. 4b shows cumulative distributions for the same four metrics as in Fig. 4a, but split into early and late phases of initial task learning and of the first rule reversal. As animals became familiar with the task (upper two rows of Fig. 4b), they began to run more slowly, choose targets later, correct their paths more often, and lick for reward later in error trials (Kolmogorov-Smirnov tests; **Early trials** of ΔOri = 30° or easier: df = 426 based on 428 trials in 5 animals; Reaction times: K-S statistic = 0.19; Path surplus: K-S = 0.26; Lick location: K-S = 0.32; Running speed: K-S = 0.06; **Late trials** of ΔOri = 30° or easier: df = 400 based on 402 trials; Reaction times: K-S = 0.25; Path surplus: K-S = 0.22; Lick location: K-S = 0.20; Running speed: K-S = 0.15; for p-values see Fig. 3a). After the rule was reversed, this behavioural pattern was transiently reversed as well in the case of path surplus and running speed, and diminished in the case of reaction times and lick location (for an example of the same reversal in an individual animal, see Supplementary Fig. S6). As animals became familiar with the new task rule, behavioural differences settled back into the original pattern, suggesting that animals began to predict the reversed outcomes correctly (Fig. 4b, third and fourth row; Kolmogorov-Smirnov tests; **Early trials** of ΔOri = 30° or easier: df = 360, based on 362 trials in 5 animals; Reaction times: K-S statistic = 0.09; Path surplus: K-S = 0.18; Lick location: K-S = 0.17; Running speed: K-S = 0.13; **Late trials** of ΔOri = 30° or easier: df = 350 based on 352 trials; Reaction times: K-S = 0.20; Path surplus: K-S = 0.29; Lick location: K-S = 0.22; Running speed: K-S = 0.09; see Supplementary Fig. S6 for the same pattern in the second rule reversal). The fact that behavioural differences between correct and incorrect trials were amplified by task training, but reversed during rule reversal, suggests strongly that they are directly related to the error predictions animals were making based on acquired task rules. In other words, animals seemed to identify potentially incorrect decisions as they were happening, leading to slower decision making, more frequent mid-trial target changes and decreased reward anticipation.

**Figure 5:**
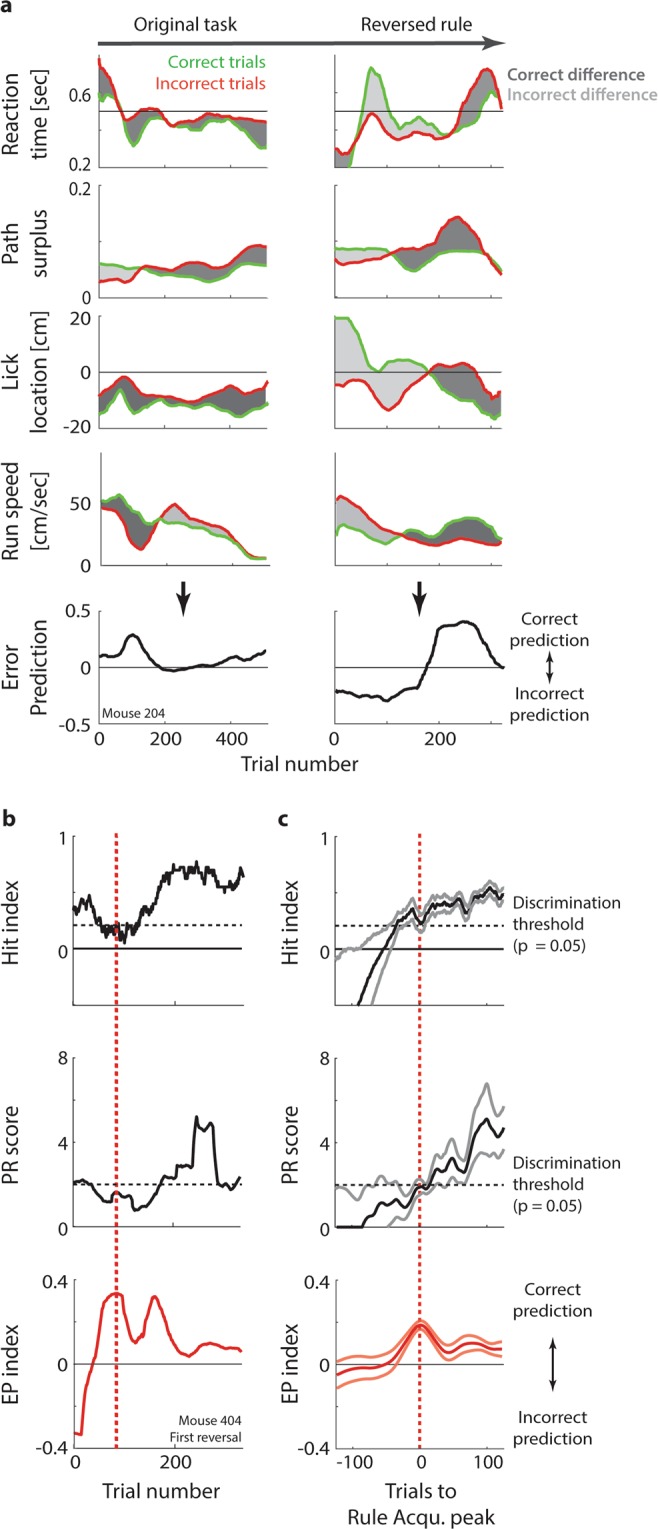
Rule learning precedes rule performance. (**a**) Top panels: Running average of reaction times (sliding window: ±25 trials) for correct trials (green) and incorrect trials (red) across original task (left) and the first rule reversal (right), for one animal. Dark grey shading: ‘Correct’ reaction time difference between correct and incorrect trials (reaction times are slower in incorrect trials than correct trials, as predicted in Fig. 4a). Light grey shading: Opposite reaction time difference (reaction times are slower in correct trials). Second row: Same for path surplus. Third row: Same for lick position. Fourth row: Same for running speed. Bottom row: Error Prediction Index, computed by averaging the normalized differences between reaction times, path surplus, lick position and running speed for correct and incorrect trials (see Methods). Positive values indicate that an animal reacts slower, takes less direct paths, licks later and runs more slowly in incorrect trials, suggesting that they correctly predict the trial to be erroneous. (**b**) Top panel: Black line: Running average of hit index (sliding window: ±25 trials) throughout training stages 5 and 6 for one animal in the first rule reversal. Dashed black line: Discrimination threshold (α = 0.05; see Fig. 3a). Second row: Same for PR score. Bottom row: Same for Error Prediction index. Red dashed line: Estimated point of rule learning, based on the first peak in the error prediction index. (**c**) Top panel: Black line: Running average of hit index (sliding window: ±25 trials) throughout rule learning (n = 15, i.e. five animals in the original task and two rule reversals). The running average is aligned to the estimated point of rule learning determined by the first peak in the Error Prediction index (see Fig. 4b and bottom panel), showing 125 trials before and after the peak. Grey lines: Average ± SEM. Black dashed line: Discrimination threshold (α = 0.05; see Fig. 2d). Red dashed line: Error prediction peak. Centre panel: Same for PR score. Bottom panel: Same for Error Prediction index. Red: Average: Light red: Average ± SEM.

Since performance differences between correct and incorrect trials seemed to be a reliable correlate of rule acquisition, we used them to create the error prediction (EP) index. By tracking ongoing behavioural differences between correct and incorrect trials, the EP index estimates an animal’s ability to predict trial outcomes throughout training. It is computed with a sliding-window analysis, averaging the normalized differences between reaction times, path surplus, running speed and lick location for correct and incorrect trials (Fig. 5a; see Methods; for an illustration of the computation using single-trial examples, see Supplementary Fig. S5). As the example in Fig. 5a demonstrates, rule reversals initially tended to evoke inversed error predictions (until ~ trial 175 in Fig. 5a, right panels), quickly followed by a peak in correct error prediction (around trial 190; for further examples, see Supplementary Fig. S7). Given how suddenly error prediction can switch from incorrect to correct, one could describe this as the equivalent of the human ‘Aha’ or ‘Heureka’ moment^65^. Note that at this point, animals do not seem to simply abandon the old task rule in favour of random/non-existent trial expectations (which would lead to an EP index close to 0), but convert to an active anticipation of the new task rule, as shown by the strong positive peak in the EP index (see Supplementary Fig. S7 for details).

Interestingly, task performance tended to improve mainly *after* the EP index had adjusted to the new task rule. Figure 5b relates the time course of the EP index to hit index and PR score in one animal. In this case, the ‘Heureka’ moment (i.e. the first peak in correct error prediction) occurred around trial 90, about 50 trials *before* task performance improved significantly. Supplementary Figure S7 shows additional examples demonstrating that this pattern applied both to initial task training and rule reversals.

To confirm that correct error prediction generally preceded correct task performance, we averaged the time courses of hit index and PR score centred around the first peak of the EP index (Fig. 5c; pooled for five animals across original task and two rule reversals). Particularly the PR score, but also the hit index, rose above the discrimination threshold only *after* error prediction had peaked. This finding suggests that animals commonly experienced a phase of task learning at which they anticipated trial outcomes correctly, but did not consistently implement this knowledge in their response, leading to low task performance.

**Figure 6:**
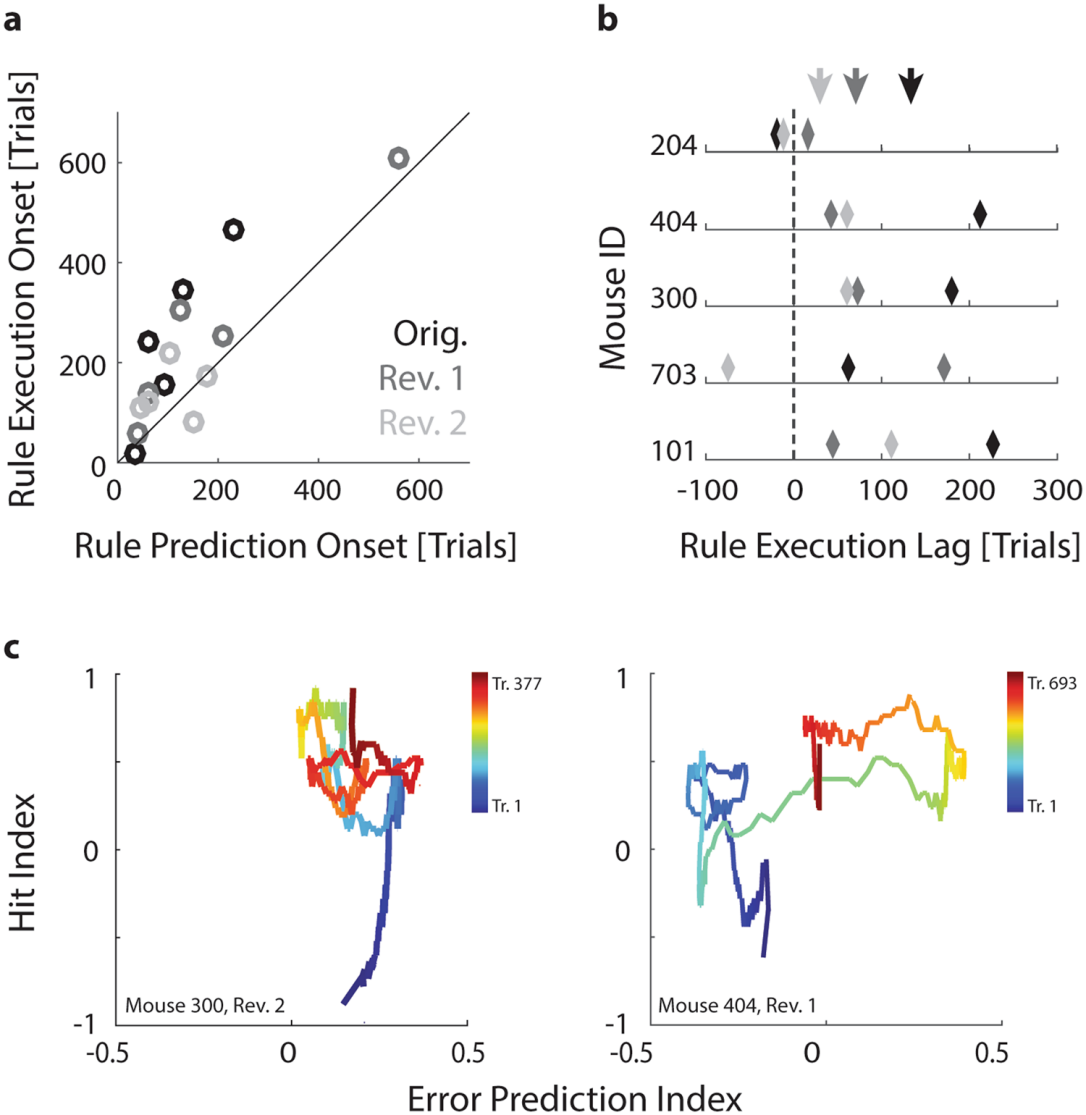
The lag between rule learning and execution varies across animals. (**a**) Relation between the onset of rule prediction (first peak in Error Prediction index) and the onset of rule execution (estimated by combining the time points at which hit index, target distance and PR score exceed their respective critical thresholds) for five animals across original task (black data points), first reversal (dark grey) and second reversal (light grey). Note that data points are concentrated above the diagonal. (**b**) Lag between the onset of rule prediction and rule execution (see panel a) for all five animals in original task (black diamonds), first reversal (dark grey diamonds) and second reversal (light grey diamonds). Arrows: Mean lag across animals for original task (black) and two reversals (dark and light grey). Dashed line: Zero lag. (**c**) Left panel: Relationship between Error Prediction index (indicating rule acquisition) and hit index (indicating successful rule execution) for one animal across the course of the second rule reversal training. Trials later in the training are drawn in warmer colours. Right panel: Same for another animal in the first rule reversal training. In the example on the left, rule prediction markedly precedes rule execution, while on the right, rule prediction and execution improve largely synchronously.

To further examine to what extent correct rule prediction preceded rule execution, we first estimated the moment of rule acquisition as the point of the first peak in the EP index for all five animals across both rule learning and reversals. We then estimated the corresponding moment of successful rule execution as the trial in which task performance exceeded the criterion values that we had previously established for the computation of visual discrimination thresholds (see Fig. 3). More specifically, we determined the respective trials in which hit index, target distance and PR score exceeded their respective bootstrapped criterion values (see Methods), and then defined the onset of rule execution as the average of these three estimates. Figure 6a shows the lag between the onset of rule prediction and rule execution (n = 5 animals in original task and two reversals). The vast majority of data points populates the triangle above the diagonal, indicating that rule prediction generally preceded rule execution by anything from a few to several hundred trials. We refer to this delay between rule prediction and rule execution as the rule execution lag.

To test whether particular rule execution lags were typical of specific animals, we plotted the lags across original task and rule reversals for each animal (Fig. 6b). While the number of animals studied here is obviously too small to identify phenotype clusters, it is interesting to note that Mouse 204 seems to execute rules immediately upon acquiring them, while Mouse 404, 300 and 703 show a reasonably long rule execution lag during initial task learning, but speed up noticeably during subsequent rule reversals. Such different styles of rule acquisition can also be visualized by plotting trajectories relating the EP index and hit index throughout the training process. Figure 6c shows two such trajectories, exemplifying two different learning styles: In the left panel, the animal already begins the training with a largely correct rule prediction, while execution catches up during the first ~90 trials. In contrast, in the right panel, the animal initially shows both incorrect rule prediction and incorrect execution, which are then corrected largely simultaneously beginning at trial ~300.

**Figure 7:**
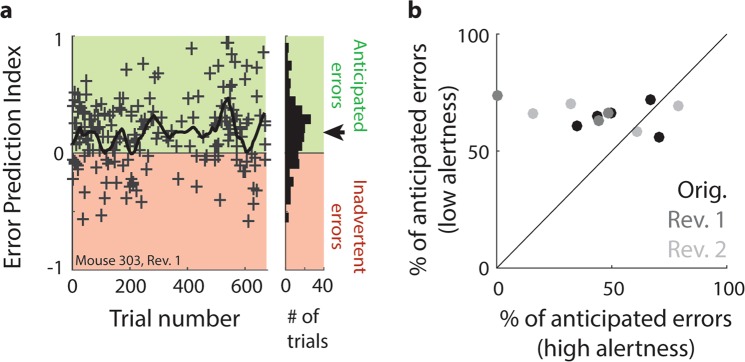
Individual errors can be identified as anticipated or inadvertent. (**a**) Left: Example of the classification process for individual error trials across the first reversal training of one animal. Each error trial is assigned an Error Prediction (EP) index based on the normalized difference in reaction time, path surplus, running speed and lick location compared to the surrounding ± 10 hit trials. Crosses: EP index for one individual error trial. Errors with an EP index > 0 (green background shade) are defined as anticipated, errors with an EP index < 0 (red background shade) as inadvertent. Right-hand inset: Distribution of EP indices across the course of training. Black arrow: Mean EP index. (**b**) Relation between the proportion of anticipated errors made during spontaneous states of high and low alertness (as defined by local PR scores, see Methods), respectively. Shown are five animals in the original task (black dots) and two reversals (dark and light grey dots, respectively), with three measurements missing due to an insufficient overall number (n < 5) of error trials. Data points are concentrated above the diagonal (*p < 0.05; based on t-test for dependent samples), indicating that the proportion of anticipated errors is significantly smaller during high than low alertness.

To go one step further in identifying the cognitive processes that generate individual error trials, we additionally tried to estimate whether each error trial was more likely to have been anticipated or inadvertent. To do so, we used a variation of the EP index which also compares the reaction time, path surplus, running speed and lick position associated with correct and incorrect trials. The difference was that here, instead of comparing sliding averages of correct and incorrect trials, we compared each individual error trial to the surrounding ± 10 correct trials (see Methods). In this way, we aimed to infer which trial outcome an animal most likely anticipated in each error trial: If the EP index computed for a specific error trial was positive, it indicated that the trial featured later decision making, more hesitant target approach and/or less anticipatory licking than in the surrounding correct trials. We would therefore classify such an error as anticipated. If on the other hand an animal showed fast decision making and anticipatory licking in an error trial, leading to a negative EP index, we assumed that the animal expected its target choice to be correct, and therefore labelled the error trial as inadvertent. Figure 7a shows the classification for all error trials completed by one animal during the first rule reversal. While especially at the start of reversal training, there are some inadvertent errors, the majority of errors is ultimately anticipated correctly (see distribution in the right-hand inset of Fig. 7a; t-test for difference from zero: df = 183 trials; t = 8.0; p < 0.001). Nevertheless, both anticipated and inadvertent errors continued to occur throughout the training process.

At this point, it is important to note that we can of course not directly verify whether our classification is correct for each individual error trial. For instance, an animal may anticipate being correct in an error trial, but lick late for reward nonetheless because it is satiated. This issue is reduced by the fact that we use a conjunction of four metrics (reaction time, path surplus, lick position and running speed) to estimate the EP index, so that e.g. late licking alone would only impact the error classification to a limited extent. To further validate our error classification, we examined its relation to another feature of behaviour that was estimated independently: We explored to what extent anticipated and inadvertent errors were likely to occur during phases of high and low alertness.

While inadvertent errors are by definition difficult to prevent, it stands to reason that anticipated errors can in principle be avoided if the animal invests additional cognitive resources into decision making and motor planning in trials in which it is uncertain of its choice. We therefore predicted that the number of anticipated errors should decrease during phases of high alertness, when such additional cognitive resources are available. In contrast, inadvertent errors should largely remain, since the animal genuinely anticipates these choices to be correct. To test this hypothesis, we first computed an estimate of spontaneous states of high and low alertness based on ongoing fluctuations of the PR score (see Methods and^49^). As shown in more detail in^49^, the PR score tended to rhythmically fluctuate throughout the course of a training session, leading to an overall bimodal distribution (for example distributions, see Supplementary Fig. S8). When such rhythmic fluctuations were related to changes in other primary performance metrics, it became clear that during phases when the PR score was high, animals also showed faster reaction times, earlier licking, faster running speeds and more efficient running trajectories (i.e. a smaller path surplus). While this has so far not been validated by more direct measures of alertness like pupil size^66–68^, the overall behavioural pattern we observed suggests that the PR score could be used to delineate phases of high and low overall performance capacity, which corresponds well to the concept of alertness (for details on this procedure and in-depth analyses of spontaneous fluctuations in task performance, see^49^).

Based on the classification of spontaneous high- and low-alert states, we then computed the proportion of anticipated errors during high and low alertness, respectively (Fig. 7b). As predicted, the proportion of anticipated errors tended to be smaller in high-alert than in low-alert states, as shown by the fact that data points in Fig. 7b are concentrated above the diagonal. This suggests that during high-alert states, animals mostly committed inadvertent errors (for individual examples linking high alertness to low error anticipation on a trial-by-trial basis, see Supplementary Fig. S8).

Note that the EP index and the PR score (used to classify high- and low-alert states) are not computationally dependent in any way – while high-alert phases generally go along with faster reaction times, shorter running paths, faster running and earlier licking (see^49^), there is no mathematical reason to expect that error trials would feature even faster reaction times, shorter paths, faster running and earlier licking than the surrounding hit trials, which would generally also be part of the same high-alert episode. The fact that the anticipation of individual error trials shows a systematic relation to the ongoing fluctuations in the level of alertness despite being computationally independent suggests that our error classification yields valid results that can be relied on as the basis for further investigations.

## Discussion

The VEF task is a novel vision-based paradigm for rule reversal learning in head-fixed mice, which meets several goals not fully achieved by previous paradigms. First, it features drastically shortened training times and minimal (15%) training drop-out, even for challenging stimulus conditions and repeated rule changes. This prevents overtraining and selection bias due to training drop-out, and opens up new options for experimental designs featuring mouse behaviour (e.g. in young animals). The task is also easily combined with various techniques of neuronal population recording and manipulation, including electrophysiology, two-photon imaging and optogenetics. Finally, it yields a concise and extensive trial-by-trial characterization of the associated learning process, allowing for direct links between ongoing neuronal activity and cognitive processing.

Capitalizing on these features, we show that mice can learn repeated vision-based rule reversals faster than expected from previous paradigms^5,7,33,39,44,45^. We also demonstrate that correct rule prediction, estimated based on a combination of behavioural markers, can precede correct rule execution by up to several hundred trials, making rule execution an uncertain proxy of rule acquisition. Finally, we use our single-trial estimates of error sources to distinguish anticipated errors from inadvertent ones, and show that anticipated errors specifically decrease when the animal is highly alert.

In terms of training efficacy, the VEF task improves substantially on comparable vision-based rule reversal tasks for mice: Training is shortened by 70–90% compared to popular touchscreen tests of flexible rule learning see also^33,39,44,45^ (3–5 versus 15–40 training sessions; but see^46,69,70^ for recent studies showing similar learning speeds in visual discrimination tasks without rule reversal – note that all of these tasks take place on a treadmill, and two out of three involve a virtual environment). This not only allows for more versatile experimental designs in time-sensitive contexts (e.g. developmental research questions or time-limited recording techniques like two-photon imaging), it also suggests that the learning process evoked by the VEF task is more akin to that encountered in other mammals such as humans and non-human primates^13–15^.

Nevertheless, the VEF task still requires more training than some of the more naturalistic paradigms: While each rule switch here takes several hundred trials to be executed correctly, mice can switch rules within a few trials in more naturalistic, and particularly in non-visual, tasks^2,32,35,37^. However, while such tasks excel at training efficiency, there is a price to pay in terms of performance quantification: Animals only complete a few dozen trials per session, limiting subsequent analyses; naturalistic responses like digging for a food reward are incompatible with head fixation, complicating neuronal recording and manipulation; sensory stimulation can neither be controlled nor recorded with precision (e.g. which objects are in the animal’s visual field at each moment), making it impossible to track the contribution of sensory input to neuronal responses throughout a trial; and task responses are quantified with similar uncertainty: Often, target choices are recorded manually, introducing observer-dependent variability and prohibiting the registration of exact response timing or ongoing behaviour (e.g. sniffing and gradual approach towards a rewarded target) as opposed to pre-determined ‘benchmark events’ (e.g. the onset of digging for a reward). Together, these limitations make it difficult to accurately characterize learning processes or relate them to ongoing neuronal activity. The VEF task trades a reasonably small increase in training time for a significant increase in stimulus control and moment-by-moment response quantification, while also being more comparable to tasks applied in humans and primates.

One of the main advantages of the VEF task lies in the fact that each trial yields a rich set of behavioural markers, which can be related to ongoing physiological and neuronal processes not only with better precision, but in a qualitatively different way. We discuss this argument in detail in^49^ with regard to the measurement of reaction times. Here, this principle can be handily illustrated with the error classification shown in Fig. 7: Our single-trial estimates of error sources take into account the speed with which animals chose their target (reaction time), the decisiveness and speed with which they subsequently pursued it (path surplus and running speed) and their anticipation of reward once they reached it (lick position). In this way, we were able to generate a plausible and validated (Fig. 7) estimate of whether an individual error trial is likely to have occurred with or without the animal’s anticipation. It stands to reason that the neural mechanisms producing anticipated and inadvertent errors could be quite different, for instance involving top-down feedback to visual cortex in the first case, but mainly feed-forward visual processing in the latter. By giving a single-trial classification of different error sources, based on the continuous registration of behaviour throughout the trial, our paradigm brings the study of these interacting but clearly distinct neural mechanisms within reach.

The behavioural metrics we extracted not only serve to quantify task performance in individual trials; they also allow us to dissect out overarching cognitive processes that contribute continuously to performance. For instance, by tracking how decisively animals approached targets and how much anticipatory licking they engaged in across correct and incorrect trials, we were able to estimate their choice confidence. This in turn allowed us to show that the more familiar animals were with the task rule, the less confidently they acted in incorrect trials. While in principle such behavioural differences between correct and incorrect trials could also be explained by fluctuating attention or by overall familiarity with the setup, the fact that behavioural differences increased with training, but transiently reversed during rule reversal, strongly favours the interpretation that they are a reflection of rule acquisition. If behavioural differences between correct and incorrect trials relied on either fluctuating attention or overall task familiarity, rule learning and especially rule reversal should only affect them minimally (for a detailed description and analysis of these scenarios, see Results). By comparing the confidence with which animals chose correct and incorrect targets throughout training, we were therefore able to estimate the emergence of correct rule predictions independently of task performance.

The estimates of rule acquisition obtained in this way suggest that when learning new rules, animals often seem to experience a sudden switch towards correct reward expectation not unlike the human ‘Aha’ moment^65^ - as evidenced by a large positive peak in the EP index. This is a fascinating finding in its own right, but also has important implications for how we measure rule learning in rodents: Virtually all currently available tests of flexible rule learning operationalize task acquisition simply as increased task performance. Specifically, the moment of rule acquisition is usually defined based on a binary hit/miss classification of individual trials, which is then compared to a pre-determined performance criterion (e.g. 18/20 correct consecutive trials). Error trials are then often labelled post-hoc as perseverative (errors occurring before criterion performance is reached) or regressive (errors occurring after criterion performance is reached)^2,4,5,32^.

Our analyses suggest that this approach misses out on several important features of rule and reversal learning in mice. First, increased task performance does not seem to be a particularly good approximation of the time of rule acquisition, since the EP index suggests that animals grasp new rules quite some time before they can successfully implement them (see^71^ for a recent similar finding in a licking paradigm). Studies that define rule acquisition based on rule execution therefore introduce a sizeable, and indeterminate, lag into subsequent analyses. Second, the delay between rule acquisition and implementation varies widely across animals, spanning anywhere from a dozen to hundreds of trials. As a result, attempting to decipher correlates of rule acquisition by aligning data from different animals onto the onset of correct rule execution will most likely ‘smear out’ such correlates substantially. Finally, the use of fixed criterion points (e.g. 85% correct performance) to define the onset of correct rule execution introduces an additional layer of confounds. For instance, out of two animals that acquire a task rule at the same time, the animal with lower overall task performance (e.g. due to impaired sensory acuity or low alertness) would usually be assigned a later onset of rule acquisition. The VEF task avoids this problem by tailoring the criterion for rule acquisition to each animal, searching for the first peak of correct rule prediction irrespective of its height or timing (in the same way, phases of high and low alertness are defined based on each animal’s individual distribution of PR scores^49^). As a consequence, both the moment of rule acquisition, and the resulting classification of perseverative and regressive errors, can be determined in a more data-driven way for each animal.

This leaves the question why a delay between correct rule prediction and rule execution should occur at all. In principle, both the presented stimuli and the required responses are highly familiar to the animal at the moment of rule reversal. As such, one might assume that once an animal understands that it simply needs to reverse the association between the already-familiar stimuli and responses, such a switch should be implemented easily and quickly – as indeed seems to be the case in higher mammals^13–15^. Why might this be different in mice?

One potential explanation can be gleaned from recent studies on task learning in mice: There is mounting evidence that after initial task learning, task execution seems to quickly be delegated to reflexive, subcortical processing routes (see e.g.^72,73^). This implies that even when a rule reversal is implemented on the cortical level, resulting in a switch in reward expectations, it might nevertheless take time and practice to rewrite the corresponding response associations, which are at that point already mediated by subcortical pathways. This may prove particularly time-consuming because animals need to both inhibit attraction to previously rewarded stimuli and to overcome avoidance of previously punished stimuli – likely requiring the modification of two at least partially separate response pathways^74,75^. The tricky interplay between cortical and subcortical processing pathways might also help to explain more generally why mice show slower (visual) reversal learning than e.g. cats^76^, non-human primates^13^ and humans^14,15^. Another potential contributing factor could be that task performance in mice tends to deteriorate considerably when animals are frustrated (see Supplementary Note S1^57,71^). Thus, even if an animal begins to understand the reversed task rule and is therefore able to correctly predict trial outcomes, the frustration associated with having experienced a large number of incorrect trials following rule reversal may nevertheless cause the animal to execute the new task erratically.

Being able to characterize rule acquisition and rule implementation separately also opens up several interesting avenues for behavioural phenotyping: First, it is possible to classify animals based on the speed with which they can put an acquired rule into action. As Fig. 6 shows, different animals seem to show a large, and quite possibly stable (i.e. trait-based) variability in their capacity to implement newly learned rules. Another interesting application is the study of rule breaking. Rule breaking is a defining component of several mental disorders, e.g. Conduct Disorder, whose underlying mechanisms could be examined using mouse models of disease^77,78^. Yet rule breaking in mice can usually not be distinguished from simple failed rule learning, which would be a mark of very different behavioural traits (e.g. impaired memory or lack of behavioural flexibility). The VEF task offers the option of identifying animals that underperform despite being able to anticipate trial outcomes correctly.

The task features discussed above open the door to a more direct translation of rule learning paradigms between species. For instance, in tasks for humans, subjects are quite routinely asked to rate the certainty with which they made a particular choice or perceived a particular stimulus. Such paradigms have been utilized e.g. to dissociate neuronal signatures of awareness from those of attention^62,63^, to infer learning algorithms employed by the brain in stochastic environments^59,60^, and to explore mechanisms of metacognition during decision making^61^. To our knowledge, our analyses of rule acquisition and of choice confidence in individual error trials are the first successful attempt to implement a similar principle in mice. In rats, a measure of choice confidence has previously been implemented using temporal wagering^64^. In this paradigm, reward is delayed by variable delays after the animal makes a target choice. The amount of time the animal is willing to wait can then be used as an estimate of the confidence with which it expects to be rewarded. While this paradigm seems to work extremely well in rats, we believe that our implementation may be more suited for mice: First, mice tend to get frustrated more easily by waiting periods and underperform quite severely as a result (^57,71^, see also Supplementary Note S1). Second, by relying on four largely independent aspects of behaviour (reaction time, path surplus, running speed and lick location), our approach allows for a more detailed and robust estimate of choice confidence not just across a session, but within a single trial. As such, the VEF task offers promising avenues for translational research into cognitive processes that had so far remained part of the behavioural ‘black box’ in mice.

Together, these insights highlight the importance of utilizing behavioural metrics that go beyond binary hit/miss classifications, as well as analysis criteria that take into account inter-individual differences: The dynamics of rule acquisition discussed above would not be visible based on global performance measures like hit rate (or even reaction time) and on fixed, universal criterion points. The reason we were able to detect them is by relying on a conjunction of non-binary single-trial performance markers; and by using a data-driven, individualized, criterion for rule learning per animal. In our view, providing behavioural metrics sensitive enough to identify and dissociate super-imposed cognitive processes and behavioural states (e.g. rule learning, alertness and perceptual difficulty) is a crucial step^29–31^ towards making sense of neuronal circuit dynamics and their contribution to behavioural decision making^79–84^.

## Methods

Data were collected from 5 male wild-type mice (Strain: C57-BL6, Charles River). All animal procedures were approved by the Ethical Committee on Animal Experimentation of Radboud University Nijmegen (RU-DEC) and the Dutch Ethical Committee on Animal Experimentation, and in accordance with the EU guidelines for animal experimentation.

### Surgical procedures

To implant the head-plate needed for head fixation, anaesthesia was induced using isoflurane, and maintained with a mixture of Ketamine (Eurovet Animal Health; Bladel, Netherlands; 75 mg/kg), Dextomidor (Zoetis; Parsippany, US; 0.5 mg/kg) and saline (0.4 ml/kg), injected intraperitoneally (2.1 ml/kg). A circular aluminum head-plate (0.2 g) was then implanted in the following steps: A circular portion of skin was removed on top of the skull, and the surrounding skin was glued to the skull with small amounts of superglue. The skull was then cleaned of tissue using a scalpel blade, and the head-plate was attached using Superbond (Sun Medical Co., Ltd; Moriyama, Japan). General analgesia during and after surgery was ensured by adding Rimadyl (Carpofen 5% with Ethanol 10%; Pfizer; New York, US; 60 mg/l) to the drinking water 2 days prior to surgery until 5 days post-surgery. In addition, local analgesia during the surgery was provided by treating exposed skin with lidocaine (‘EMLA’) cream (AstraZeneca; London, UK). After surgery, animals were injected with Antisedan (Zoetis; Parsippany, US), diluted in saline 1:10 (5 mg/kg).

### Behavioural setup

Mice were head-fixed atop a treadmill consisting of a styrofoam ball (Graham Sweet Studios; Cardiff, UK; diameter 20 cm) floating in a custom mould developed in-house (Radboud University Technocenter). The surrounding virtual environment was projected onto a spherical screen (Fibresports UK; Basildon, UK; diameter 136 cm) covering 270° of visual angle, using a projector (Optoma X501; Optoma; Fremont, US) positioned behind the screen and a spherical mirror located underneath the treadmill (diameter 38 cm; see Fig. 1a). Mice were head-fixed by attaching two fixation holders to the hinges of an implanted head-plate (see Supplementary Movie S1; head-plate design by Jasper Poort, University College London; head plates and fixation holders produced in-house; all components supporting the dome, treadmill and head holders by Thorlabs; Dachau/Munich, Germany).

To capture locomotion, two computer mice (Logitech G500; Newark, US) placed orthogonally to each other along the horizontal axis of the treadmill registered forward and lateral movement, respectively. Readouts were recorded at a frequency of 60 Hz using custom Python scripts integrated in the virtual environment (programmed in Blender; www.blender.org) and adapted from the Gnoom platform by Christoph Schmidt-Hieber (https://research.pasteur.fr/en/software/gnoom/).

Liquid reward was delivered through a tube in front of the animal’s mouth (built in-house). The tube was opened and closed by a valve (NResearch Inc.; West Caldwell, US) controlled by an Arduino Duemilanove board (Arduino; Turin, Italy) connected to the virtual environment. A lick sensor (built in-house) was integrated in the tube holder, and provided an analogue measurement of licking activity, which was recorded with a sampling rate of 60 Hz via a second Arduino Duemilanove board, and stored together with the locomotion traces. The lick sensor itself was based on simple circuit closing: A ground wire was connected to the animal via one of the head holders. At the same time, the metal holder containing the reward tube provided an analogue input to a second Arduino Duemilanove board. Whenever the animal made a connection to the reward tube, e.g. by touching the outside of the tube, or the reward liquid, the circuit was closed, sending the recorded signal sharply to zero. Unlike movement/vibration or beam-breaking sensors, this sensor did not need to be calibrated and did not miss licks even when mechanical movement was minimal.

### Task structure

While different stages of training differed in specifics like target position (see below), a training session generally adhered to the following structure: One hour before the session, mice were water deprived. They were then head-fixed on the treadmill and trained in a series of progressively more complex tasks, navigating a virtual environment based on visual cues. To succeed in a trial, animals had to run through a wall displaying the target grating. There were two different types of error trials: Animals could either not run through any wall, or in advanced training stages (5–7), they could run through the wall displaying a distractor grating.

When a trial was completed successfully, the animal heard a ‘reward’ tone and received a liquid reward of 10 μl of sweetened soy milk, dispensed from the tube in front of its mouth. In case of error trials, animals would enter a virtual ‘time-out’ corridor – a dimly lit corridor they had to traverse in order to initiate the next trial. The previous trial was then re-initiated. If animals in later training stages not only missed the target, but navigated towards the distractor, they would additionally hear a ‘punishment’ tone of loud white noise. If animals failed at the same trial more than once, they would receive gentle manual guidance towards the correct target. This guidance could take somewhat different forms (see Supplementary Movie S1) depending on how each animal responded. Note that repeat trials were not analysed since the animal’s performance depended on the previous trial (for example, some animals learned to automatically move to the opposite side after an error trial) as well as potential manual guidance from the experimenter.

The final task was structured as follows: Animals were initially presented with a grey target wall located in the centre of the virtual environment. Once the animal moved towards the target, it would cross an invisible trigger threshold, causing the target to move either to the left (40% of trials), centre (20%) or right (40%), and display a circular sinusoidal grating. Centre trials simply required the animal to keep running straight ahead and were not analysed further. When targets moved to the side, a distractor target simultaneously moved to the contralateral location and displayed a competing grating of different orientation. In the original task, targets displayed gratings oriented more horizontally, while distractors displayed gratings oriented more vertically. The easiest discrimination trials thus featured a horizontal target and vertical distractor (90° orientation difference), while the hardest discrimination featured a 42.5° target and a 47.5° distractor (5° difference). A schematic of the task is shown in Fig. 1b (see also Supplementary Movie S1). Subsequent rule reversals exchanged the identity of rewarded and punished stimuli.

### Stimulus presentation

For the final task, the virtual environment consisted of a grey-scale space with a dark floor and a light sky (both 75% contrast), with the horizon positioned ~5° visual angle above the azimuth. At each trial start, a grey (50% contrast) wall with a size of ~15 × 15° visual angle appeared at the centre of the visual field. Once animals crossed the invisible trigger zone (see ‘Task Structure’ section above and Fig. 1b), two square grey walls moved 45° to the left and right and displayed the target or distractor, respectively. Both target and distractor consisted of circular, sinusoidal gratings displayed at 100% contrast, but with different orientations (see ‘Task Structure’). Directly after the target shift, the gratings had a spatial frequency of 4.5°/cycle, and both the gratings and the square walls they were displayed on had a size of ~18 × 18° visual angle. Subsequently, both stimuli of course changed size, location etc. based on the animal’s locomotion.

### Pre-training protocol

Before head-plate implantation, mice were housed in groups in an enriched environment (High Makrolon® cages with Enviro Dri® bedding material and Mouse Igloo®) and had free access to dry food and water. After head-plate implantation, mice were kept overnight in a warming chamber (38.5 °C) for recovery, and then transitioned to individual housing with a reversed 12–12 day-night cycle (sunrise at 8 pm) and an added running wheel in the cage (Fast-Tracs®). Mice were allowed at least one week of recovery, with intermittent social contact (approximately every 2 days) with littermates. 3–5 days prior to training, animals were put on a restricted food schedule designed to reduce their body weight by 20%. Mice received 2.0–2.5 g of dry food per day. When they reached 75–80% of their initial weight, they were habituated to handling by being put on a soft cloth, repeatedly immobilised gently for a few seconds and then fed with liquid reward from a pipette. The liquid reward both for handling and training consisted of 15.5 g powdered baby soymilk (SMA Nutrition, Gatwick, UK) and 2 g of sugar per 100 ml of water. Task training began when animals stopped showing signs of distress when being handled, often actively seeking out the hand of the experimenter. This was generally the case after 1–4 handling sessions.

### Training protocol

Mice were housed individually on a reversed light cycle (Lights on from 8 pm to 8 am), and were trained in the afternoon (~2–6 pm). We trained 2–4 animals per day, in the same sequence, so that each animal had a fixed training time. Every animal completed one training session per day, which typically lasted ~45–60 minutes (minimally 20 minutes, maximally 90 minutes). A session was terminated before 60 minutes if either the number of licks or the number of correctly initiated trials dropped, signalling fatigue. A session was extended beyond 60 minutes if an animal was still licking for reward and approaching targets efficiently, and if a new training stage had been introduced recently. Depending on their performance, animals could progress through up to three training stages per session (see next paragraph). After training, animals would receive a performance-dependent reward (pieces of nuts and raisins) on the experimenter’s hand. They would then get ‘play time’ with litter mates that were being trained on the same day, before being returned to their home cage, where they received dry food pellets at least 30 minutes after training had concluded.

The behavioural training leading up to the discrimination task consisted of seven consecutive stages, which were designed based on seven inter-related principles we have found to facilitate behavioural training in mice (see Supplementary Note 1). The training steps were:

(A) In the first training stage, animals were placed on the treadmill in the dark to help them adjust to the new environment. The animal is rewarded every time it makes a few steps forwards. When animals stopped showing signs of stress and were able to move forwards they were moved to next training stage.
  (B) This training stage features a dimly lit corridor in which low-contrast target gratings appear at increasing distances. Targets always displayed a horizontal sinusoidal grating to associate horizontal orientation with reward. Target walls fill the entire corridor, making it impossible for animals to avoid them. Whenever the animal moved through a target wall, a reward was delivered. Animals quickly learned to anticipate rewards, as evidenced by them beginning to lick when approaching a target (Supplementary Fig. S1c). When a mouse learned to walk independently and lick when reward was delivered (signalled by a clicking tone of the valve and the drop of soy milk touching the animal’s mouth), the second training stage began. This generally happened within ~5–30 minutes.
The second training stage resembles stage 1B, except that the corridor surrounding the target walls was removed. Opening up the virtual space allows animals to miss the target, in which case they would not receive a reward. After a few seconds of running, the next target wall would then appear. In this way, animals learned to navigate actively towards the target to gain reward. When animals had a success rate >=80%, they were moved to training stage 3.
In training stage 3, targets are initially located in the centre of the virtual environment, but move backwards to the left or right at a 45° angle when the animal crosses an invisible trigger threshold located several steps in front of the initial target location. If animals did not approach the centre target, the trial was reset immediately. Thus, the animal initiates the trial by running directly towards the centre target. Animals tended to immediately attempt to follow targets, suggesting that they already treated rewarded targets as approachable objects in regular space. As such, training stages 3 mainly serves to train the motor skills needed to steer easily in all directions. Side biases are also tackled in this step: Targets move to one side until animals achieve two consecutive correct trials, then switch to the other side (see Supplementary Fig. S1d,e for an analysis of the decrease in side bias throughout training). In this way, animals could not obtain reward when they ignored trials on one side. When animals reached a success rate of >=80% for both target locations, they advanced to training stage 4.
Training stage 4 was the first training step to represent a simplified version of the final task. As such, targets were initially located in the centre as in stage 3, but could now move to the left (40% of trials), right (40% of trials) as well as backwards (20% of trials). Trials were pseudo-randomized, evening out over 10 consecutive trials. Most importantly, a ‘weak’ distractor stimulus in the form of a low-contrast vertical grating moving in the opposite direction of the target was added in trials when the target was moving to the left or right. When animals chose to approach the distractor, they experienced punishment in form of an auditory white noise stimulus and having to run through a ‘time-out’ corridor (see above). In this way, animals learned to ignore distractors while continuing to pursue the targets they had previously been trained on. It was crucial to move on from this training step as soon as animals learned to choose the target over the distractor (>=80% success rate) in order to avoid associating reward with differing stimulus contrasts rather than differing grating orientations.
Training step 5 was the same as training step 4, except that distractors now displayed vertical sinusoidal gratings at the same contrast as the targets. For some animals, step 5 followed seamlessly from step 4, while for others the equal grating contrast initially impaired the discrimination between target and distractor, and required more frequent manual guidance after error trials.
In the final training step, trials with progressively smaller orientation difference between target and distractor were added gradually. While the original target and distractor had an orientation of 0° and 90°, respectively, trials were progressively added in which the orientation of both target and distractor approached 45°. This gave rise to a maximum of eight orientation differences randomly interleaved in a training session: 90° (target 0°; distractor 90°); 70° (target 10°; distractor 80°); 50° (target 20°; distractor 70°); 30° (target 30°; distractor 60°); 20° (target 35°; distractor 55°); 15° (target 37.5°; distractor 52.5°); 10° (target 40°; distractor 50°); and 5° (target 42.5°; distractor 47.5°). Each trial difficulty was added when animals had adapted to the previous one to the extent that their success rate in the most difficult trials was >=70%. In some cases, animals stopped initiating trials when the trial difficulty was raised at this speed. In those cases, we decreased difficulty by one step until the animal was initiating trials and licking for reward at its usual rate. Note that not all animals reached the most difficult task conditions – for some animals, overall performance and trial initiation decreased steeply when trials with 5° or 10° orientation differences were introduced. In those cases, we removed the more difficult trials and set 15°/10° as the maximum difficulty. This training step could be implemented immediately and with minimal loss of task performance once animals mastered step 5, suggesting that at this point animals were easily able to generalize the task rule of ‘Steer towards the more horizontal stimulus.’
To train reversed task rules, steps 4–6 would be repeated with inversed target identities (e.g. vertical targets and horizontal distractors rather than vice versa).

Note that in training stages 4–7, centre trials did not require a perceptual decision, and were not analysed further. Rather, they served two purposes in terms of training: They provided a baseline number of trials in which animals were highly likely to be rewarded, heightening motivation; and they prevented animals from acquiring a habit of slowing down before every target shift in anticipation of having to change direction.

Manual guidance was used throughout training, but particularly at early training stages, in order to speed up the learning process (see Supplementary Movie S1). In training stage 1, manual guidance was used to get animals to start walking if they were sitting still frequently, as well as to prevent them from walking backwards or spinning sideways on the ball. In training stages 2–7, manual guidance was only given in the repeat trials following an error trial. As a result, guided trials were not included in the subsequent data analysis. In training stages 2–3, manual guidance tended to be more involved, since animals still needed to learn how to steer. In training stages 4–5, animals usually only required a small nudge in the correct direction once in a while. By training stage 6, animals were usually able to complete the task entirely without assistance, and manual guidance was only used to avoid frustration if an animal failed a trial more than once. During reversal learning, manual guidance in post-error repeat trials again became necessary more frequently particularly during early training, in order to help animals overcome their learned avoidance of the new target (i.e. the previous distractor).

### Data collection

Behavioural data were recorded at a sampling rate of 60 Hz using custom Python scripts, which were adapted from the Gnoom platform provided by Christoph Schmidt-Hieber (https://research.pasteur.fr/en/software/gnoom/) and integrated in the virtual environment programmed in Blender (www.blender.org). The analogue readouts of forward and lateral movement were translated into locomotion within the virtual environment while also being recorded in a text file. The corresponding lateral (X) and longitudinal (Y) position of the animal within the virtual environment were recorded at the same time. In addition, a lick sensor (see above) provided another analogue input, which was read and stored together with the other readouts.

The translation factor of actual locomotion to movement within the virtual environment was 3.0, i.e. animals ran three times the distance they traversed in the virtual environment. The reason was that mice actually ran so fast on the treadmill that once targets came close enough to be perceived at all, animals would already have run past the target before they could even begin to change running direction. In rare cases during the third training stage (introduction of lateral target positions), we increased the lateral gain transiently by up to 20% when animals struggled to reach lateral targets (see Supplementary Fig. S1a).

Data were stored in two separate text files: One contained the time stamps of discrete events per trial generated within the game (trial onset, trial offset, time of target shift), as well as some simple behavioural variables per trial (target reached or missed, amount of reward received). The second file contained a continuous 60-Hz read-out of behavioural measurements, specifically locomotion and licking behaviour. Finally, training sessions were regularly filmed with a small webcam (Logitech C310; Logitech; Newark, US) positioned at the right-hand corner of the virtual environment dome (see Supplementary Movie S1).

### Primary performance metrics

Data were analysed using custom scripts written in Matlab (Mathworks; Natick, US). The recordings of locomotion, virtual position and licking were first cut into trials, then we extracted seven primary performance metrics per trial (e.g. reaction times). These primary metrics were defined and measured as follows:

#### Hit index

Trial success was classified as 1 when the animal touched the target, −1 when it touched the distractor, and 0 when it touched neither. Since this metric is not identical to the classical hit rate, we refer to it as hit index.

Note that for training stage 3 (targets moving to the side without a distractor), we already encoded trials in which the animal moved to the opposite side of the target (where the distractor would later be positioned) as −1 rather than 0. This was done in order to make a distinction between near-misses and trials in which the animal was not aiming for the target at all. Wide misses occurred particularly if animals initially showed a side bias, causing them to run to one side irrespective of where the target was positioned. Such biases generally resolved themselves within a few dozen trials (see Supplementary Fig. S1; also see the training progressions shown in Supplementary Figs S2 and S4 – in both cases, training stage 3 begins with a negative hit index, most likely indicating a side bias, but transitions to a positive hit index within 20–50 trials).

#### Target Distance

The target distance is a parametric measure of accuracy, and is defined as the lateral distance between the animal and the target at trial offset. It was computed in the following way:

1 $$TD=|\frac{{X}_{T}-{X}_{M}}{{\Delta }{X}_{T}}|$$

where TD is the target distance, X_T_ is the lateral (X) position of the target edge closest to the animal, X_M_ is the lateral (X) position of the animal at the longitudinal (Y) position level with the target, and ΔX_T_ is the distance between two adjacent target positions.

The distance between the animal and the target is normalized by the distance between two adjacent target positions (ΔX_T_) in order to make the resulting values comparable across specific task implementations: Irrespective of the exact spatial layout of the virtual environment, a target distance of 1 denotes that by the end of the trial, the animal was so far removed from the target that it could have touched another target position. For example, if the target appeared on the left then the animal running straight ahead would result in a target distance ~1, whereas if the animal ran to the right, the target distance would be ~2. Note that all correct trials result in a target distance of 0.

#### Path reliability (PR) score

The PR score is the third metric of accuracy, assessing the spatial precision with which animals aim for each target position. It is computed by comparing running paths across multiple trials (see Fig. 2c). To do so, we first created a standardized representation of each running path: For Y positions starting at the trigger zone and moving in steps of 2 cm up to the target position, we computed the average lateral (X) position for each trial’s running trajectory. For each point in Y, we then computed the discriminability between the average X positions for running paths in which targets were positioned on the left and right, respectively. Discriminability was quantified using Cohen’s D^51^, which normalizes the difference between two averages by their associated pooled standard deviations:

2 $${D}_{y}=\frac{|X{L}_{y}-X{R}_{y}|}{\sqrt{\frac{({n}_{L}-1)\cdot {s}_{L,y}^{2}+({n}_{R}-1)\cdot {s}_{R,y}^{2}\,}{{n}_{L}+{n}_{R}-2}}}$$

where D_y_ is the discriminability at longitudinal position Y, XL_y_ is the mean X position at position y across all running paths for which the target is located on the left, XR_y_ is the same for targets located on the right, n_L_ is the number of trials with targets on the left, n_R_ is the number of trials with targets on the right, s_L,y_ is the standard deviation of all X positions for position Y with the target on the left, and s_R,y_ is the same for targets on the right. This yielded a vector of discriminability values across Y space, as shown in Fig. 2c. The maximum of this vector (e.g. ~2.9 in Fig. 2c) was used to represent the PR score for a trial.

We computed the PR score as a global metric across all trials in a session, as a stimulus-dependent metric for the trials of each stimulus difficulty (see Fig. 3), and as a local running average per trial (applied in Fig. 7b). In the latter case, for each trial we computed the local PR score by taking into account a total of 15 trials, i.e. 7 trials prior to and 7 trials following the trial in question.

The PR score decreases when the number of incorrect trials increases, because incorrect paths increase the standard deviation of X positions. Conversely, it increases when running trajectories towards the same target location not only touch the target but do so in a spatially identical (i.e. replicable) way, decreasing the standard deviation. Note that centre trials were not included in the computation of the PR score since they did not require an active target choice.

#### Path surplus

The path surplus quantifies whether animals take the shortest route towards the target or make additional direction changes. It mainly increases when an animal ‘changes its mind’ and changes running directions midway to a target location. To compute the path surplus, the length of the animal’s running path from the location where it makes its target choice (estimated based on the reaction time) to the target location is compared with an ideal path length. The ideal path length is computed as the Euclidean distance between the animal’s position at the moment of target choice, and the centre of the target:

3 $${L}_{i}=\sqrt{{({X}_{T}-{X}_{RT})}^{2}+{({Y}_{T}-{Y}_{RT})}^{2}}$$

where L_i_ is the ideal path length, X_T_ is the position of the target centre in X, Y_T_ is the position of the target centre in Y, X_RT_ is the X position of the animal at the point of the reaction time, and Y_RT_ is the equivalent in Y. The actual path length was then computed as follows:

4 $${L}_{R}={\sum }_{t=1}^{t=n-1}\sqrt{{({x}_{t+1}-{x}_{t})}^{2}+{({y}_{t+1}-{y}_{t})}^{2}\,}$$

where L_R_ is the actual path length, t denotes all consecutive measurements of x and y locations, beginning from the point of the target choice (t = 1) until one data point before the trial end (t = n − 1). Finally, the path surplus PS was computed as:

5 $$PS=\frac{{L}_{R}}{{L}_{I}}-1$$

Thus, a path surplus of 0 would signify that the actual path was as long as the ideal one, while a path surplus of 0.5 would indicate that the actual path was 50% longer than the ideal one. A path surplus < 0 could occasionally occur because the ideal path length was computed using the position of the target centre, whereas animals might hit the closest edge of the target, shortening the path slightly compared to the ‘ideal path’. Note that the path surplus does not take into account the correctness of the target choice (since this is already reflected in target distance, hit index, and PR score) – it only measures how efficiently animals move from the point of target choice towards the target they are closest to at the end of the trial. In other words, we took into account the target location that the animal approached, whether correctly or incorrectly.

#### Reaction time

For all trials in which targets moved to the side, we computed a reaction time based on the change in running direction that animals initiated after the target shift. The computation took into account running trajectories from 0.5 seconds before the target shift to 3 seconds after the target shift, and identified the timing of the largest change in running direction within that window.

Naturally, the running direction at a specific time point t needs to be determined based on the animal’s displacement in a time window surrounding that time point. We define this time window as τ:

6 $$\tau =[t-T,\,t+T]$$

where t is a time point (i.e. sampling point of the recording) and T is a fixed interval determining the size of the averaging window. Longer time windows T will filter out ‘noise’ (e.g. based on a step the animal made) but will also overlook sudden changes in running direction. We therefore initially computed reaction time estimates using T values of 2, 5, 10, 15, and 25 sampling points, corresponding to 33, 83, 167, 250 and 417 ms (given a 60 Hz acquisition rate). We found that T = 10 (i.e. a window of 20 sampling points, or 333 ms) yielded robust estimates that also best represented the joint results of all other T values.

To estimate direction changes robustly given the low number of data points in each trial (3.5 seconds of running trajectory × 60 Hz sampling frequency = 210 samples per trial), we were keen to minimize the number of parameters fitted to the running trajectories. We therefore computed a linear regression between two partially overlapping portions x_τ_ and x_τ+Δ_ of the animal’s running path:

7 $${x}_{\tau +{\rm{\Delta }}}={b}_{t}\cdot {x}_{\tau }+{m}_{t}+{{\epsilon }}_{t}$$

where x_τ_ is an animal’s lateral positions x in the virtual environment over time window τ, x_τ+Δ_ is a vector of lateral positions x of the same size, but shifted forwards in time by Δ; b_t_ is the slope of the function converting x_τ_ to x_τ+Δ_ for time point t, m_t_ is the offset of the function at time point t, and ε_t_ is the corresponding error term. For each t value, we fitted a linear regression function between all x values in the window τ and all x values in window τ + Δ (see Equation 6). The linear regression function was fitted in Matlab using the criterion of least square errors, i.e. minimizing ε^2^. After exploring Δ values of 1 to 15 sampling points, we found that an overlap of 5 sampling points, i.e. 83 ms (or 25% of the 20-sampling-point window) worked well to identify the biggest changes in running direction. We then used the slope b_t_ to identify direction changes. If b_t_ at time point t was close to 1, it indicated that the animal was showing the same lateral movement in both portions of the running path, i.e. running in the same direction. In contrast, slopes higher or lower than 1 would indicate changes in running direction. As a result, the change in running direction could directly be estimated without first estimating the running direction of each path portion separately, effectively halving the number of parameters that needed to be fitted.

Based on the vector of slope estimates b_t_ for all time points (see Fig. 2b), we defined the reaction time as the moment at which the slope most differed from 1, limiting the range of possible reaction times to 0.1–1.25 seconds. Reaction times < 0.1 seconds were physiologically unlikely, and reaction times >1.25 seconds indicated trials in which the animal was not focused on the task for some reason. If b_t_ differed from 1 by < 0.1, this indicated that the animal had not substantially changed its running direction (e.g. because it was already running to one side before the target moved). In those trials reaction times were not defined.

#### Running speed

The mean running speed for a trial was computed by averaging the running speeds starting at the target shift up to the point when the animal was within 10 cm of the target. We used this analysis window in order to capture the animal’s true response speed: Prior to the target shift (i.e. before crossing the trigger zone), running speed tended to be shaped by the previous trial, e.g. when animals were still licking for reward from the previous target. On the other hand, some animals would slow down close to the target to start licking for reward. Including this portion of the trial would obscure the speed with which the animal approached the target.

#### Lick position

To assess whether animals were licking more in anticipation of or in response to reward, we quantified at which longitudinal (Y) location animals were licking (see Figs 2b and S1c). For each trial, we took into account the Y position of the target ±30 cm. Since the space between the animal’s starting position and the target had a length of 62 cm, this means that we considered approximately the last half of the space leading up to the target, and the first half of the following trial’s space. The average lick position per trial was then computed as the mean of the included lick positions relative to the target position.

#### Secondary analyses of performance

From these primary performance indicators, we generated six secondary metrics, which are discussed in detail in^49^. For the purposes of the current study, the following secondary analyses were relevant:

#### Visual discrimination thresholds

To measure each animal’s perceptual threshold for orientation discrimination, we first generated psychophysical curves of hit index, target distance and PR score as a function of ΔOri (Fig. 3a). Using the ‘fit’ function in Matlab, we fitted the psychophysical curves with a logistic function (Fig. 3):

8 $$f({\rm{\Delta }}\mathrm{Ori})=\frac{L}{1+{e}^{-s\ast ({\rm{\Delta }}\mathrm{Ori}-o)}}+\varepsilon $$

where f(ΔOri) is the observed psychophysical curve f as a function of the orientation difference ΔOri, L is the maximum value of the curve, s is the steepness of the curve, o is the horizontal offset of the curve, and ε is the error term between the sigmoid function and the observed psychophysical curve, with the fitting algorithm minimizing ε.

For the fitted functions, a discrimination threshold was defined as the ΔOri for which the function reached its criterion value. For the hit index, the criterion was 0.2 (with 1 representing perfect performance and 0 representing chance); For target distance, the criterion was 0.82 (with 0 representing perfect performance, and 1 representing chance); For the PR score, the criterion was 1.25 (with scores >3 representing largely perfect performance, and scores <1 representing chance).

The criterion values were determined as follows: In order to decide whether a particular outcome (e.g. a hit index of 0.27) represents chance or above-chance visual discrimination, we need to know the error variance associated with such an outcome. Since psychophysical curves consist of the average performance per ΔOri, the relevant measure of error variance is the standard error of the mean (SEM) rather than the standard deviation. To estimate the SEM for hit index, target distance and PR score, respectively, we relied on a bootstrapping procedure: For each ΔOri, we repeatedly randomly sampled 20, 40, 60, and 80% of trials and computed the resulting SEM as SD/√n, where n is the number of trials (10 repetitions for each ΔOri and trial fraction combination). This procedure was repeated for each animal, and all resulting SEMs were pooled. We then used the mean of the pooled SEM distribution as the criterion threshold for non-random performance. We chose to use the mean of the SEM distribution (rather than e.g. the 95^th^ percentile) because a perceptual threshold is generally taken to reflect the point at which there is a 50% probability of correct performance. The average SEM should represent that case closely – given that SEMs were bootstrapped by sub-sampling trial numbers, performance at the criterion value should be significantly above chance in more than 50% of tests. For the hit index, the average SEM was 0.19, leading to a criterion of 0.2; For target distance, the average SEM was 0.17, leading to a criterion of 0.82; For the PR score the average SEM was 1.20, leading to a criterion of 1.25.

#### Error Prediction (EP) index

The EP index combines the normalized differences between reaction times, path surplus, running speed and lick position in correct trials versus incorrect trials (Figs 5–7). The normalized difference for reaction times, path surplus and lick position was computed as follows:

9 $${\rm{\Delta }}P=\frac{{P}_{Miss}-{P}_{Hit}}{abs({P}_{Miss})+abs({P}_{Hit})}$$

where ΔP is the normalized difference, P_Hit_ is the average performance (reaction time, path surplus, running speed or lick position) in correct trials, and P_Miss_ is the corresponding average performance in incorrect trials. For running speed, the difference between P_miss_ and P_Hit_ was computed in the opposite direction, subtracting P_miss_ from P_Hit_. Note that ΔP can in principle vary between −1 and 1, and takes on positive values when animals react slower/run less efficiently/run slower/lick for reward later in incorrect than in correct trials, indicating accurate error prediction. The EP index is then the average of the four measures:

10 $$EP=\,({\rm{\Delta }}RT+{\rm{\Delta }}PS+{\rm{\Delta }}RS+{\rm{\Delta }}LP)/4$$

where EP is the error prediction index, ΔRT is the normalized difference in reaction times, ΔPS is the normalized difference in path surplus, ΔRS is the normalized difference in running speed and ΔLP is the normalized difference in lick positions. The EP index can therefore in theory take on values between −1 and 1, with positive values indicating correct error prediction. In practice, EP indices <−0.75 and >0.75 were not observed.

Like the PR score, the EP index can be computed for different groups of trials. In principle, it can be computed across the entire performance of an animal or a training session, and would then reflect the general ability to predict error trials. Here, we did not apply the EP index as such an overall performance measure. Instead, we used a sliding-window analysis of the EP index in order to track the changing error predictions of animals throughout the learning process (see Figs 5 and 6). Since animals generally performed the task with high accuracy, comparing the same number of correct and incorrect trials would have resulted in comparing a small time window of clustered hit trials to a large time window of scattered error trials – which could introduce spurious differences due to performance variations over time. To avoid this issue, we computed each sliding EP index by comparing 20 consecutive incorrect trials with all the correct trials interleaved between them (usually a higher number). Finally, for the error-source analysis of individual error trials shown in Fig. 7, we computed a single-trial EP index, which compared one individual error trial with the surrounding ±10 correct trials.

Note that in the case of missing values (e.g. because a reaction time could not be defined in a specific trial, or an animal did not lick for reward, resulting in a non-defined lick location), a trial was nevertheless included in the computation of the EP index as long as at least two out of the four constituting primary metrics were defined. The EP index was then simply computed based on all available primary measurements.

#### Rule prediction onset

The onset of correct rule prediction was determined as the trial at which the sliding EP index (see preceding paragraph) reached its first positive peak. Since the first peak was generally very obvious (see examples in Figs 5b and S7), we determined its position by eye (Fig. 6).

#### Rule execution onset

We estimated the onset of successful rule execution as the trial in which performance accuracy (measured by hit index, target distance and PR score) exceeded the criterion values previously established for the computation of visual discrimination thresholds (see beginning of this section and Fig. 3). Specifically, we created smoothed vectors of hit index, target distance and PR score using a sliding 20-trial averaging window, and determined the respective trials in which these smoothed vectors first exceeded their bootstrapped criterion values (see beginning of this section) (Fig. 6). The rule execution onset was then defined as the average of these three onset estimates.

#### Spontaneous states of high and low alertness, defined by the bimodal distribution of local PR scores

Local PR scores were bi-modally distributed in the vast majority (>90%) of sessions (see^49^ for extensive analysis) (Figs 7 and S8). To identify the cut-off point between the two modes of a local PR score distribution, we used the approach proposed by^85^. This analysis simply compares the amount of variance in two portions of a distribution with the total variance across the entire distribution:

11 $$F=\frac{Va{r}_{All}}{mean(Va{r}_{part1},\,Va{r}_{part2})}$$

where Var_All_ is the total variance across the distribution and Var_part1_ and Var_Part2_ are the variances in the parts of the distribution above and below the cut-off point, respectively. The distribution is repeatedly cut in two at different points, and the cut-off point between the two modes is then determined as the one that results in the largest F value. After determining an individual cut-off point for every animal, trials with a local PR score larger than this cut-off point were considered High-Alert trials, the rest as Low-Alert trials.

### Statistical tests

The statistical analysis of the presented results had to account for the fact that the data set included in this manuscript contained measurements from only five animals. This number does not provide sufficient statistical power to meaningfully test differences such as the ones shown in Fig. 1d (training durations across rule reversals), Fig. 3b (visual thresholds across rule reversals), Fig. 6a,b (rule execution lags across rule reversals), Figs 7b and S8c (anticipated error rate in high- versus low-alert trials across rule reversals), and Fig. S7b (EP index peak heights across rule reversals); since all these analyses would require repeated-measures ANOVAs (Figs 1d, 3b, 6b and S7b) and two-way ANOVAs (Figs 6a, 7b and S8c). We therefore opted to treat these analyses as preliminary proof-of-principle results rather than report them together with essentially uninformative statistical indicators. As a result, we only report statistical tests when they can be based on a larger sample size, as is the case for the cumulative distributions across trials shown in Fig. 4 and Supplementary Fig. S6, the EP index distribution across trials shown in Fig. 7a, correlations for 12 animals completing the original task shown in Supplementary Fig. S1b, and correlations across stimulus conditions (n = 8) shown in Supplementary Fig. S4. The statistical tests we used throughout the manuscript are as follows:

#### Kolomogorov-Smirnov test for differences between distributions

To confirm differences between the distributions of reaction times/path surplus/lick positions for early and late training, respectively, we applied a two-sample Kolmogorov-Smirnov test (Matlab function ‘kstest2’) (Figs 4, S6).

#### T-test for deviation of a distribution from zero

To test whether the distribution of single-trial EP index estimates for all error trials in the first rule reversal training of Mouse 303 significantly exceeded zero, we ran the Matlab function ‘ttest’ on the entire set of EP index values (Fig. 7a).

#### Statistical significance of correlation coefficients

The statistical significance of individual correlation coefficients was directly extracted from Matlab’s ‘corrcoef’ function (Figs S1b and S4c,d).

#### Correction for multiple comparisons

In cases when multiple statistical significances were computed, either across performance measures (Figs 4 and S3) and/or at multiple time points (Fig. 4), we evaluated individual p-values against critical α values produced by the Dunn-Sidak Correction for multiple comparisons^86^ (Figs 4, S1b, S4c,d and S6):

12 $${\alpha }_{Corr}=1-{(1-\alpha )}^{\frac{1}{n}}$$

where α_Corr_ is the corrected critical error probability based on the desired family-wise error probability α, and n is the number of independent comparisons. In our case, we computed two α_Corr_ corresponding to α = 0.05 and 0.01 (indicated with 1 and 2 stars, respectively, above Figs 4 and S6). For Figs 4 and S6b, the number of multiple comparisons was 12 (4 time points × 3 performance measures). For Fig. S6a, the number was 6 (2 time points × 3 performance measures). For Supplementary Fig. S1b, it was four correlations, and for Supplementary Fig. S4c,d, it was 18 (3 time points x 6 independent metrics – out of seven primary metrics, hit index, target distance and PR score are mathematically interdependent, leading to a conservative estimate of six actually independent metrics).

## Supplementary information

Supplementary Materials

Supplementary Movie S1

## Data Availability

The data sets and analysis tools presented in the current manuscript are available from the corresponding author on reasonable request.
