# Supplementary material for: The Virtual-Environment-Foraging Task enables rapid training and single-trial metrics of rule acquisition and reversal in head-fixed mice
Source: Sci Rep. 2019 Mar 18;9:4790. doi: 10.1038/s41598-019-41250-w (PMC6423024; doi:10.1038/s41598-019-41250-w)
Supplement: Supplementary file 1 — Supplementary Materials [file 41598_2019_41250_MOESM1_ESM.pdf]

Supplementary Materials

The Virtual-Environment-Foraging Task enables rapid training and single-trial metrics of rule acquisition and reversal in head-fixed mice

Martha N. Havenith, Peter M. Zijderveld, Sabrina van Heukelum, Shaghayegh Abghari, Paul Tiesinga, Jeffrey C. Glennon

Contents

1) Supplementary Note: Seven principles of task design for mice
2) Supplementary Figure S1: Optimizing training strategies
3) Supplementary Figure S2: Example of reversal training
4) Supplementary Figure S3: Computing reaction times
5) Supplementary Figure S4: Primary performance metrics throughout task training and reversal
6) Supplementary Figure S5: Computing the Error Prediction index
7) Supplementary Figure S6: Behavioural differences between correct and incorrect trials
8) Supplementary Figure S7: Rule prediction precedes rule execution in task learning and reversal
9) Supplementary Figure S8: Animals make more inadvertent errors during High-Alert phases
10) Supplementary Movie S1: Example of training progression

Supplementary Note: Seven principles of task design for mice

The behavioural training leading up to the task consisted of seven consecutive stages, which were designed based on seven inter-related rules we have found to facilitate behavioural training in mice (see supplementary references [1,2] below for related descriptions):

1) Motivate: The task should always be significantly more rewarding than time spent in any other context. An example of this principle is shown in Supp. Figure S1a: At the beginning of training stage 4, an animal was consistently missing the target. This might simply indicate a failure to acquire the task. However, a small manual adjustment of the head holder's position and an increase of 10% in the gain of lateral motion facilitated running on the treadmill, and this resulted in an almost immediate, marked improvement of performance. This suggests that the main factor blocking correct performance was not task comprehension, but the fact that the situation was not sufficiently rewarding overall (i.e. the animal initially had to invest too much energy per trial compared to the reward size).

2) Minimize stress: Learning can be considerably impaired when animals are stressed, even in the presence of reward ([3-5]; but see [6]). Gentle habituation is paramount, as indicated in Supp. Figure S1b: Longer delays between surgery and training, as well as prolonged handling prior to training resulted in faster task learning. Note that boosting learning speed did not equate to boosting general task performance of an animal, as evidenced by the fact that behavioural adaptation did not predict the maximal stimulus difficulty an animal was able to achieve.

3) Trade, don't punish: Following from rules 1 and 2, we found aversive punishments (e.g. air puffs) to delay learning. We therefore instated trade-offs for incorrect decisions, e.g. through a time-out corridor following error trials, as well as restarting miss trials until the

animal succeeded. Note that correction trials were not analysed. Instead, they served to reinforce an increasing price of failure in terms of running distance, and as 'instruction trials' so that animals could memorize the correct choice (Rule 4). In the same vein, the fact that changing running direction on the treadmill was somewhat energy-consuming ensured that, in contrast to easier response paradigms (e.g. licking), animals never responded spontaneously or randomly. We tested this in two animals by simply switching off the projection of the virtual environment for ~ 15 minutes. In the absence of a visible target, both mice never changed running direction abruptly (data not shown). An example of the power of (in this case unintended) trade-offs is shown in Supp. Figure S1a: The animal's initial task performance is poor, but improves almost instantly when the settings of the treadmill are marginally adjusted (lateral gain is increased by 10%) to make targets slightly easier to reach. In other words, the initial task configuration set the 'price' of successful task performance too high for this animal to make learning an attractive option.

4) Frustration leads to superstition: As shown by [7], in the absence of clear evidence, mice tend towards 'superstitious' decision-making, and stop responding to cues that could ensure success. As a consequence, for a training sequence to be successful, each training stage needs to be solvable in a majority of trials. It is also important not to entrain transient training steps for too long in order to avoid frustration about unexpected rule changes. To keep a sufficient percentage of trials solvable, we a) test a range of orientation differences (**Δ**Ori) from 90**°** to 5**°**, with easier trials serving as 'anchor trials', and b) manually guide animals towards the correct target after repeated error trials.

5) Avoid abstraction: Unsurprisingly, mice are not readily able to learn abstract associations. It is therefore important to represent conceptual associations in a physical way. For example, rewarding animals immediately upon approaching the correct target made the

reward association almost instant - animals began anticipatory licking within 50-200 trials (Supp. Fig. S1c, see also [[8]]). Given that trials in early training tended to last 2-10 seconds, this corresponds to a training time of 5-15 minutes before reward anticipation set in. Similarly, to clearly signify time-outs, we used a dedicated time-out environment. This increased learning rates markedly compared to an unmarked time countdown in a non-descript environment (data not shown).

6) Utilize innate behaviours: Following from rule 5, it is helpful to use innate behaviours to facilitate stimulus-response links. In this case, we created an environment that mimics foraging (pursuing a visual target to obtain food in a cluttered environment). It also led us to abandon go-no-go tasks, since mice do not easily inhibit behaviours like running or licking for reward.

7) Detect, don’t discriminate: Since we can assume that mice have a very limited capacity to attend to multiple objects simultaneously, choosing between two stimuli is more difficult than detecting one target from a background. Such a task still requires visual discrimination, but conceptually the animal searches for one target instead of comparing two. We therefore presented the target stimuli from the beginning, only gradually introducing distractors (initially at low contrast) as a 'cluttered background'. This approach was vastly more successful than a training scheme we attempted in two mice, where we immediately introduced both types of stimuli. In these cases, performance did not exceed chance level after three training sessions (data not shown). In other tasks this principle also has been utilized explicitly or implicitly (e.g. [[1,9]]).

References – Supplementary Note

1 Guo, Z. V. *et al.* Procedures for behavioral experiments in head-fixed mice. *PLoS One* **9**, e88678, doi:10.1371/journal.pone.0088678 (2014).

2 Burgess, C. P. *et al.* High-Yield Methods for Accurate Two-Alternative Visual Psychophysics in Head-Fixed Mice. *Cell Rep* **20**, 2513-2524, doi:10.1016/j.celrep.2017.08.047 (2017).

3 Murphy, B. L., Arnsten, A. F., Jentsch, J. D. & Roth, R. H. Dopamine and spatial working memory in rats and monkeys: pharmacological reversal of stress-induced impairment. *J Neurosci* **16**, 7768-7775 (1996).

4 Bondi, C. O., Rodriguez, G., Gould, G. G., Frazer, A. & Morilak, D. A. Chronic unpredictable stress induces a cognitive deficit and anxiety-like behavior in rats that is prevented by chronic antidepressant drug treatment. *Neuropsychopharmacology* **33**, 320-331, doi:10.1038/sj.npp.1301410 (2008).

5 Qin, S., Hermans, E. J., van Marle, H. J., Luo, J. & Fernandez, G. Acute psychological stress reduces working memory-related activity in the dorsolateral prefrontal cortex. *Biol Psychiatry* **66**, 25-32, doi:10.1016/j.biopsych.2009.03.006 (2009).

6 Graybeal, C. *et al.* Paradoxical reversal learning enhancement by stress or prefrontal cortical damage: rescue with BDNF. *Nat Neurosci* **14**, 1507-1509, doi:10.1038/nn.2954 (2011).

7 Busse, L. *et al.* The detection of visual contrast in the behaving mouse. *J Neurosci* **31**, 11351-11361, doi:10.1523/JNEUROSCI.6689-10.2011 (2011).

8 Killeen, P. R. Models of trace decay, eligibility for reinforcement, and delay of reinforcement gradients, from exponential to hyperboloid. *Behav Processes* **87**, 57-63, doi:10.1016/j.beproc.2010.12.016 (2011).

9 Pinto, L. *et al.* Fast modulation of visual perception by basal forebrain cholinergic neurons. *Nat Neurosci* **16**, 1857-1863, doi:10.1038/nn.3552 (2013).

10 Havenith, M. N. *et al.* Rapid training and single-trial metrics: A visual discrimination task measuring cued and sustained attention in head-fixed mice. *Nature Scientific Reports* (2018).

Figure S1: Optimizing training strategies

a) Top panel: Hit index for the first session of training stage 3 (moving targets, no distractors) for Mouse 702. Grey circles: Single-trial measures. Black line: 15-trial running average. Red line: Time point when we adjusted the treadmill position and increased the lateral gain by 10% (see Methods, section on Data Collection). Note the rapid increase in performance after training parameters were adjusted. Bottom panel: Same for the absolute distance from the target rather than the hit index (see Methods).

b) Top panels: Number of trials needed to reach the final training stage, as a function of, respectively, the number of days between surgery and training onset (left), and the number of handling sessions before training (right). Red circles: 12 animals trained in the original task (including the five animals that were subsequently trained on rule reversals). Asterisks: Statistical significance of correlation coefficients (* $p < 0.05$; ** $p < 0.01$). Bottom panels: Same, but for the smallest ΔOri reached. Animals that had experienced increased handling and habituation learned the task more quickly, but this was unrelated to the visual acuity or overall task performance that the animals ultimately reached. Thus, handling apparently did not affect overall ability, but enhanced the speed with which each animal reached its optimal performance.

c) Lick positions for three animals, shown from fastest (left panel) to slowest (right panel) onset of reward anticipation. Circles: Single-trial measures. Lines: 15-trial running average. Background colours denote the first three training stages (see inset). Arrows: Approximate onset of consistent reward anticipation, when the lick positions drops below the horizontal line, as determined by eye. Overall, animals

appeared to develop reward anticipation within 250 trials or less. This corresponds to a time period of approximately 10-20 minutes.

d) Running average of four representative behavioural metrics for one animal, covering the first training session in which lateral targets were introduced (i.e. training stage 3). All metrics were averaged using a 25-trial sliding averaging window. Red: Trials with target on the left. Blue: trials with target on the right. Inset numbers: Quantification of side bias at four different points in the training session.

e) Running average of side bias for the same metrics as in d across all animals for the first 250 trials following the onset of training stage 3. Coloured lines: Animals. Grey line: Mean across animals. Shade: Mean ± 1 St.Dev. The side bias was computed as a running average across small trial sets in order to test whether animals responded differently to left/right targets over time. Side bias could be estimated for each performance metric. To compute the side bias, we created separate sliding 25-trial averages of each performance metric for trials featuring left targets and right targets and then determined the normalized difference between the two vectors. The resulting side bias could take values between -1 and 1, with -1 indicating a larger metric for right-hand trials, 1 indicating a larger metric for left-hand trials, and 0 indicating balance between left and right trials (for details, see [10]). As the figure shows, left and right preferences are balanced across time and across animals, and side bias decreases over time, disappearing almost entirely within 250 trials (i.e. ~1-2 training sessions).

Figure S1

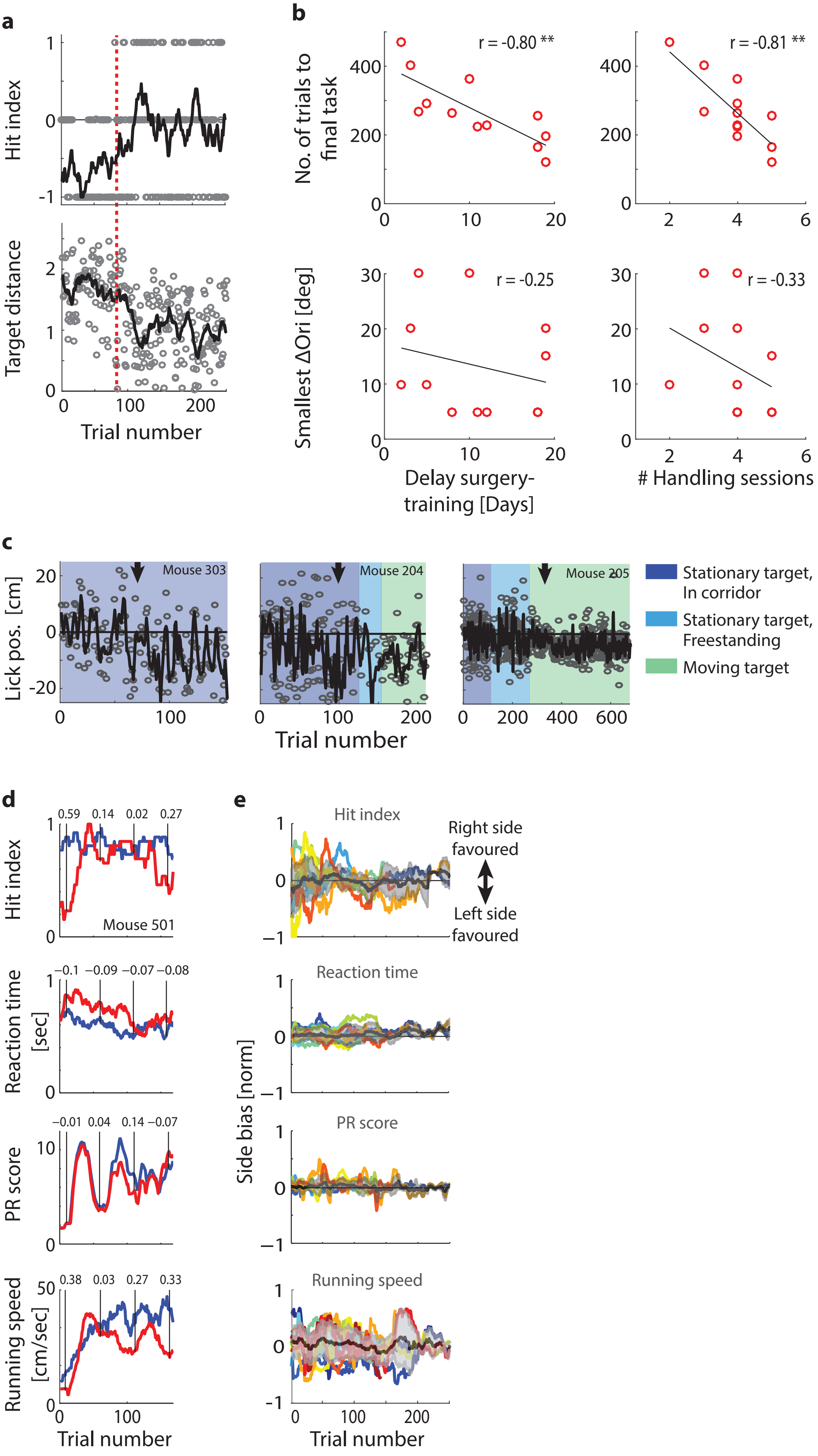
a
Hit index
Target distance
Trial number
b
No. of trials to final task
r = -0.80 **
r = -0.81 **
Smallest ΔOri [deg]
r = -0.25
r = -0.33
Delay surgery-training [Days]
Handling sessions
c
Lick pos. [cm]
Mouse 303
Mouse 204
Mouse 205
Trial number
Stationary target, In corridor
Stationary target, Freestanding
Moving target
d
Hit index
Mouse 501
Reaction time [sec]
PR score
Running speed [cm/sec]
Trial number
e
Hit index
Right side favoured
Left side favoured
Reaction time
Side bias [norm]
PR score
Running speed
Trial number

Figure S2: Example of reversal training

a) Evolution of hit index for one example animal across original task (top panel) and two rule reversals (centre and bottom panel, respectively). Data points are colour-coded by training stage (see Fig. 1c). Coloured circles: Running 10-trial average of hit index. Coloured lines: Corresponding 15-trial running average. Grey vertical lines: Transitions between training sessions. Training for reversed task rules began with a few trials of training stage 3 (moving targets, no distractors). To keep training times comparable between the original and reversed tasks, we only show the training progression from training stage 3 onwards also for the original task.

Figure S2

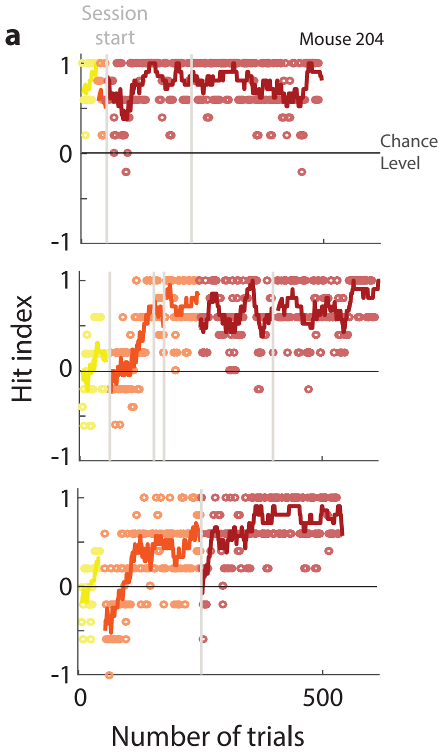
a
Session start
Mouse 204
1
0
-1
Chance Level
Hit index
1
0
-1
1
0
-1
0
500
Number of trials

Figure S3: Computing reaction times

a) Upper panel: Example running trajectory for one trial (Trial 125) taken from training session 3 of mouse 101. Black trace: The animal's lateral (X) position as a function of time. Black vertical line: Moment of target shift. Black dashed line: Measured reaction time. Green rectangles: Example of two overlapping analysis windows of the running trajectory (Width: 20 data points; Overlap: 5 data points; see Methods), which are then correlated to each other in order to measure the local change in running direction. Note that these two analysis windows are focused on parts of the running trajectory before the target shift. Red rectangles: Same as green rectangles but for portions of the running trajectory that surround the measured reaction time. Blue rectangles: Same, but for portions of the running trajectory that take place after the reaction time. Center panels: Regression between the X-position traces highlighted in the upper panel by green/red/blue rectangles. Circles: Relation between 20 x-position data points captured by, respectively, the earlier analysis window (X axis) and the later analysis window (Y axis). Black line: Fitted regression function. b: Slope of the regression function. Note that for the green and blue analysis windows, the slope is close to 1 (b = 1.50 and b= 1.37), indicating only small direction changes. In contrast, for the red analysis windows, the slope is b = -2.16, indicating a large direction change. Bottom panel: Full curve of regression slopes across the entire trial. Green, red and blue diamonds highlight the slope estimates contributed by the analysis windows shown in the preceding panels. Note that at the time point covered by the red analysis windows, which corresponds to the measured reaction time, the slope deviates sharply from 1, but not before or after.

b) Same as a for a different trial (Trial 129) of the same training session.

Figure S3

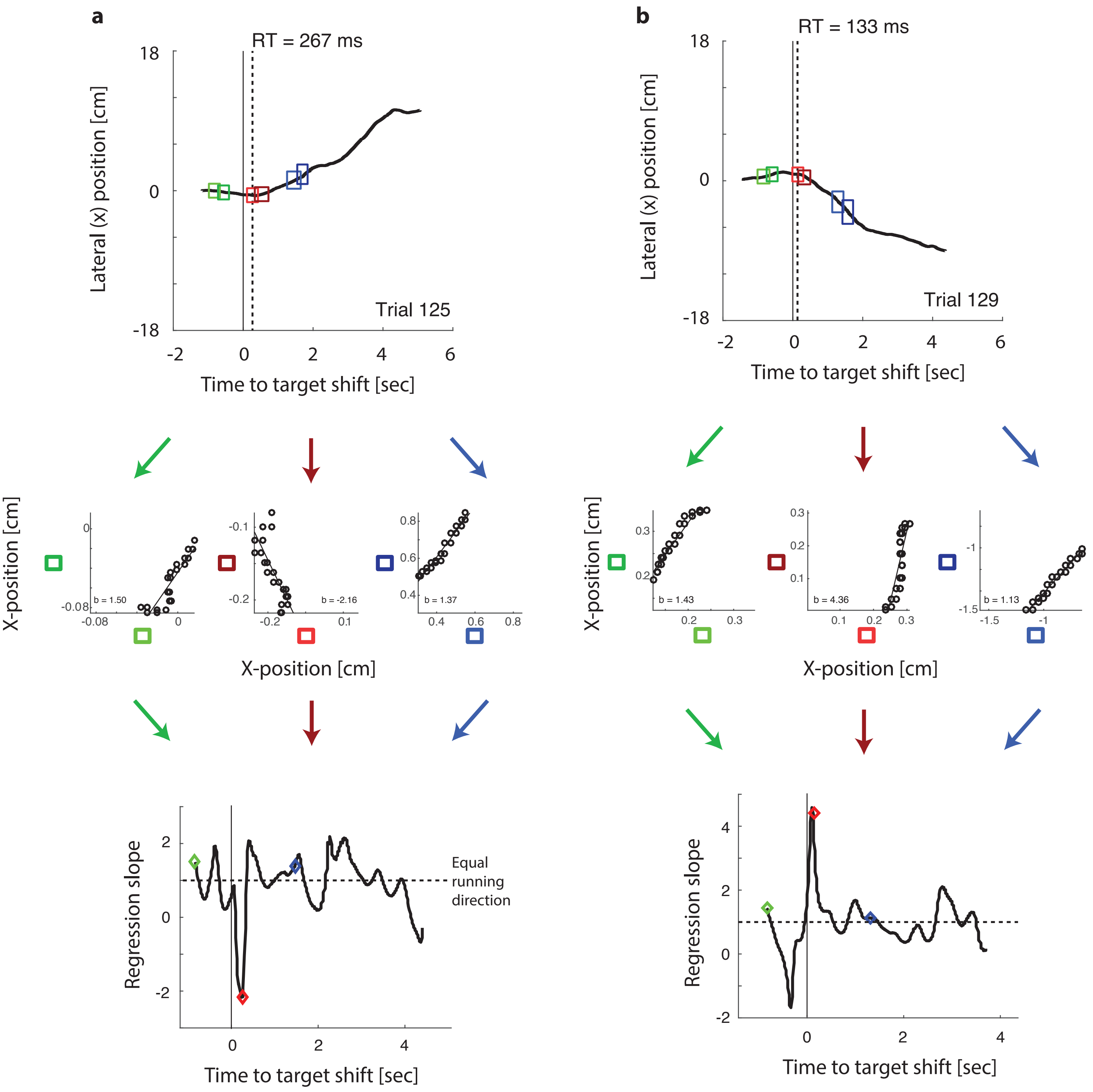

a
RT = 267 ms
Lateral (x) position [cm]
18
0
-18
-2
0
2
4
6
Trial 125
Time to target shift [sec]
X-position [cm]
b = 1.50
b = -2.16
b = 1.37
X-position [cm]
Regression slope
Equal
running
direction
Time to target shift [sec]
b
RT = 133 ms
Trial 129
Time to target shift [sec]
b = 1.43
b = 4.36
b = 1.13
X-position [cm]
Regression slope
Time to target shift [sec]

Figure S4: Primary performance metrics throughout task training and reversal

a) Primary performance metrics across the learning process of one animal, colour-coded by training stage (see Fig. 1c). Coloured circles: Single-trial metrics (except for hit index: Running average over 10 trials). Coloured lines: 15-trial running averages. Grey vertical lines: Transitions between training sessions. Black vertical lines: Points when smaller **Δ**Ori were introduced, indicated at the top of figure. Black arrow in upper panel: Range of trials used for the computation of the psychophysical curves shown in panel b.

b) Psychophysical curves of all primary metrics from the animal shown in a, as a function of **Δ**Ori. All trials following the introduction of trials with **Δ**Ori ≤ 20° were included (see black arrow in top panel of a). Line: Mean performance. Error bars: SEM.

c) Left-hand panels: Same as b, but showing psychophysical curves averaged across five animals. Centre panels: Same for the first reversal. Right-hand panels: Same for the second reversal. Insets show correlation coefficients between ΔOri and each performance metric (* family-wise $p < 0.05$; ** family-wise $p < 0.01$ after Dunn-Sidak correction for multiple comparisons; see Methods). These correlations reveal that some performance metrics, e.g. hit index and reaction time, had a largely linear relation with stimulus difficulty (ΔOri). In contrast, metrics like running speed and lick position were largely independent of stimulus difficulty. The psychophysical curves after rule reversals indicate that task performance in this context tended to be less consistently related to stimulus difficulty. This was especially true for the first reversal, while performance after the second reversal seemed to be more comparable to initial performance. The stimulus dependence of task performance after rule reversals likely decreases because at this point rule acquisition and frustration affect behavioural output more heavily, overriding

pure stimulus processing. Note that for both reversals, performance was still close to optimal.

Figure S4

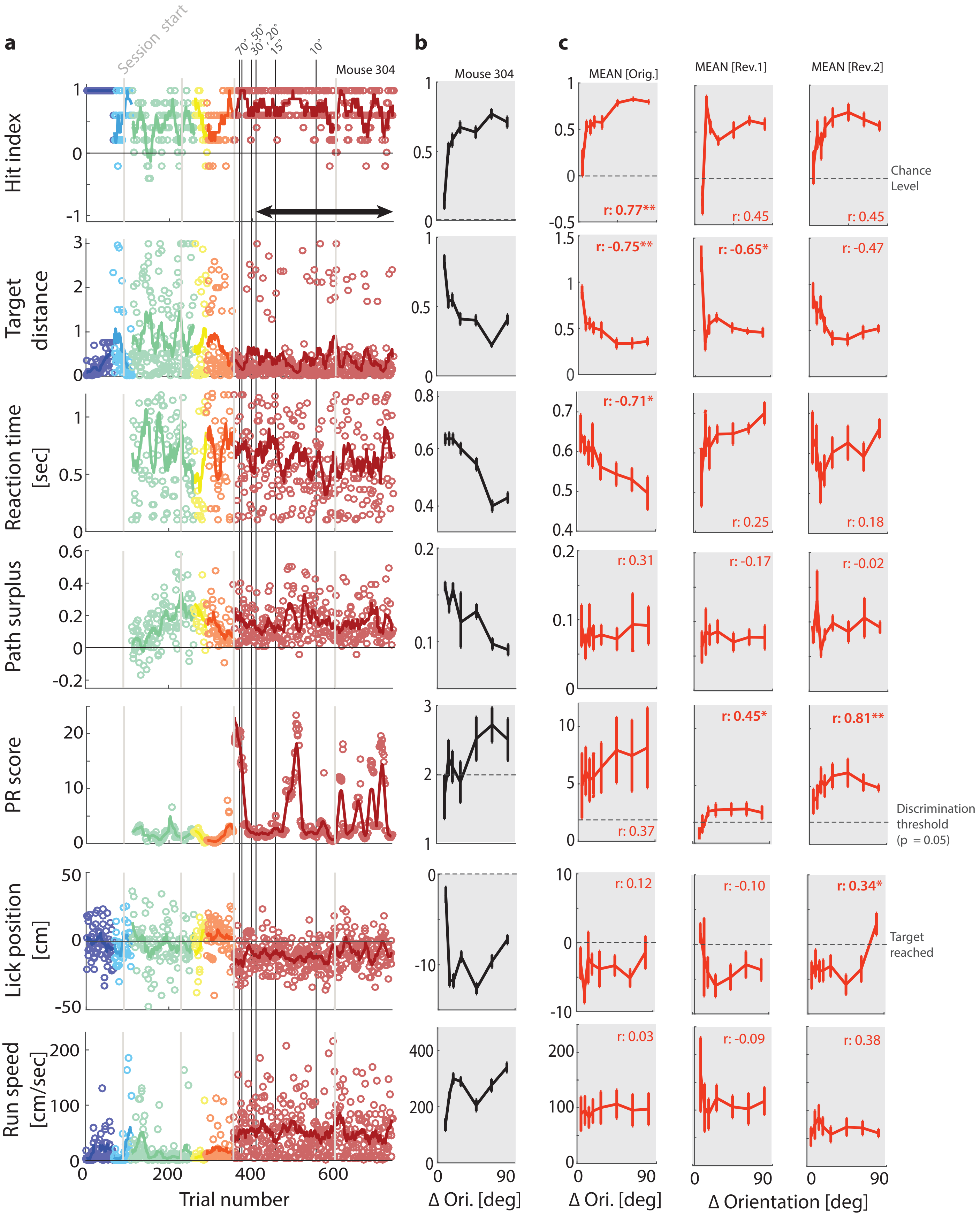
a
b
c
Session start
70°, 50°, 30°, 20°, 15°
10°
Mouse 304
Hit index
Target distance
Reaction time [sec]
Path surplus
PR score
Lick position [cm]
Run speed [cm/sec]
Trial number
Δ Ori. [deg]
MEAN [Orig.]
MEAN [Rev.1]
MEAN [Rev.2]
Chance Level
r: 0.77**
r: 0.45
r: 0.45
r: -0.75**
r: -0.65*
r: -0.47
r: -0.71*
r: 0.25
r: 0.18
r: 0.31
r: -0.17
r: -0.02
r: 0.45*
r: 0.81**
r: 0.37
Discrimination threshold (p = 0.05)
r: 0.12
r: -0.10
r: 0.34*
Target reached
r: 0.03
r: -0.09
r: 0.38
Δ Orientation [deg]

Figure S5: Computing the Error Prediction index

a) Example running trajectories, showing the animal's lateral (X) position in the virtual environment as a function of its longitudinal (Y) position for five correct (top row) and five incorrect (bottom row) trials. The trials were part of the first task reversal of mouse 101, and were chosen randomly from a range of ± 25 trials around the first peak in the EP index. Note that given the reasonably low error rates, a range of 50 trials was necessary to be able to make a random selection from a pool of error trials. Red/green trace: Running trajectory. Red/green diamonds: Licks for reward, superimposed on the running trajectory. Black vertical line: Position at which the target shift occurs. Red dashed line: Position at which the estimated reaction time occurs. Black dashed line: Longitudinal position of the target. Note that in correct trials, the lateral position of the animal is reset after reaching the target position, but in incorrect trials this is not the case. The reason is that when animals hit the target, they are immediately reset to a new trial, whereas if they fail to hit the target, they have to run towards a second reset wall positioned 20 cm behind the target. Inset text: Measured reaction time, lick position, average running speed from target shift to end of trial and path surplus based on the running and licking trajectory shown for each trial.

b) Left panel: Reaction times measured in the correct (green dots) and incorrect (red dots) trials shown in a). Black horizontal line: Average. Normalized difference is computed as $(\text{mean}_{\text{Incorrect}} - \text{mean}_{\text{Correct}}) / (|\text{mean}_{\text{Incorrect}}| + |\text{mean}_{\text{correct}}|)$. Center left panel: Same for path surplus. Center right panel: Same for lick position. Right: Same for running speed. Note that the normalized difference for running speed is computed in the opposite direction from the other metrics (subtracting incorrect from correct trials rather than vice versa). The reason is that if animals predict trial outcomes, the other metrics (reaction time, path surplus and lick position) would be expected to be smaller in correct than incorrect trials, while running speed would be expected to be greater in correct than

incorrect trials. Inset on the right: The EP index for the ten trials shown in a is computed as the average of the normalized differences shown in the four figure panels on the left. In this case, the four normalized differences of, respectively, 0.26, 0.20, 0.41 and 0.65 together result in an EP index of 0.38.

Figure S5

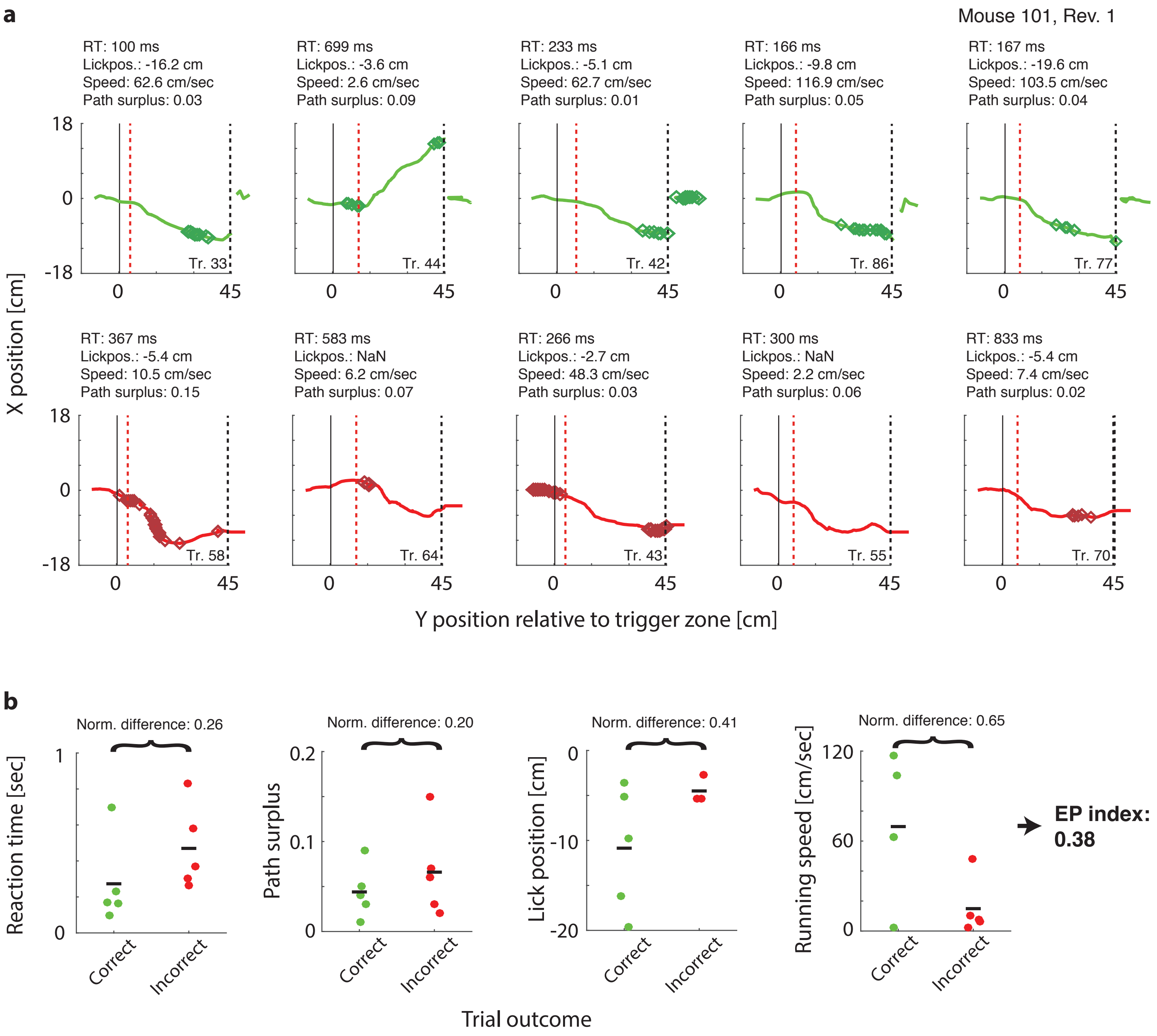

a
Mouse 101, Rev. 1
RT: 100 ms
Lickpos.: -16.2 cm
Speed: 62.6 cm/sec
Path surplus: 0.03
RT: 699 ms
Lickpos.: -3.6 cm
Speed: 2.6 cm/sec
Path surplus: 0.09
RT: 233 ms
Lickpos.: -5.1 cm
Speed: 62.7 cm/sec
Path surplus: 0.01
RT: 166 ms
Lickpos.: -9.8 cm
Speed: 116.9 cm/sec
Path surplus: 0.05
RT: 167 ms
Lickpos.: -19.6 cm
Speed: 103.5 cm/sec
Path surplus: 0.04
Tr. 33
Tr. 44
Tr. 42
Tr. 86
Tr. 77
RT: 367 ms
Lickpos.: -5.4 cm
Speed: 10.5 cm/sec
Path surplus: 0.15
RT: 583 ms
Lickpos.: NaN
Speed: 6.2 cm/sec
Path surplus: 0.07
RT: 266 ms
Lickpos.: -2.7 cm
Speed: 48.3 cm/sec
Path surplus: 0.03
RT: 300 ms
Lickpos.: NaN
Speed: 2.2 cm/sec
Path surplus: 0.06
RT: 833 ms
Lickpos.: -5.4 cm
Speed: 7.4 cm/sec
Path surplus: 0.02
Tr. 58
Tr. 64
Tr. 43
Tr. 55
Tr. 70
X position [cm]
Y position relative to trigger zone [cm]
b
Norm. difference: 0.26
Norm. difference: 0.20
Norm. difference: 0.41
Norm. difference: 0.65
Reaction time [sec]
Path surplus
Lick position [cm]
Running speed [cm/sec]
Correct
Incorrect
Trial outcome
EP index: 0.38

Figure S6: Behavioural differences between correct and incorrect trials

a) Differences in reaction time, path surplus, running speed and lick location for correct versus incorrect trials, at early and late training stages of the second task reversal. Top row: Cumulative distribution function (CDF) of reaction times (left), path surplus (centre left), lick positions (centre right) and running speed (right), for the first 30% of trials beginning at training stage 5, pooled across all animals. Green: Correct trials. Red: Incorrect trials. Insets: P-values resulting from a Kolmogorov-Smirnov test of the distribution differences between correct and incorrect trials (* $p < 0.05$; ** $p < 0.01$ after correction for multiple comparisons; see Methods; **Early trials**: df = 336 based on 338 trials from 5 animals; Reaction times K-S stat: 0.06; Path surplus K-S: 0.14; Lick position K-S: 0.23; Running speed K-S: 0.06). Second row: Same for the last 30% of trials in the second rule reversal (**Late trials**: df = 329 based on 331 trials; Reaction times K-S: 0.17; Path surplus K-S: 0.28; Lick position K-S: 0.29; Running speed K-S: 0.27). Note that differences show the same pattern as for the first rule reversal shown in Fig. 4b.

b) Same as a, but for early and late training stages of original rule acquisition and first rule reversal (see Fig.4b), and for only one example animal. Top row: Cumulative distribution function (CDF) of reaction times (left), path surplus (centre left), lick positions (centre right) and running speed (right), for the first 30% of trials beginning at training stage 5 (df = 65 based on 67 trials; Reaction times K-S stat: 0.52; Path surplus K-S: 0.39; Lick position K-S: 0.27; Running speed K-S: 0.19). Second row: Same for the last 30% of trials in the original task (df = 63 based on 65 trials; Reaction times K-S stat: 0.70; Path surplus K-S: 0.69; Lick position K-S: 0.34; Running speed K-S: 0.26). Third row: Same for the first 30% of trials after the first rule reversal (df = 61 based on 63 trials; Reaction times K-S stat: 0.27; Path surplus K-S: 0.25; Lick position K-S: 0.45; Running

speed K-S: 0.11). Grey shade in background highlights that this is the time point when distribution differences are transiently diminished and/or reversed. Bottom row: Same for the final 30% of trials after the first rule reversal (df = 59 based on 61 trials; Reaction times K-S stat: 0.42; Path surplus K-S: 0.54; Lick position K-S: 0.51; Running speed K-S: 0.32). Note that for this animal, the behavioural differences for path surplus and running speed actually reverse after the task rule is reversed, while the differences for reaction time and lick location diminish.

Figure S6

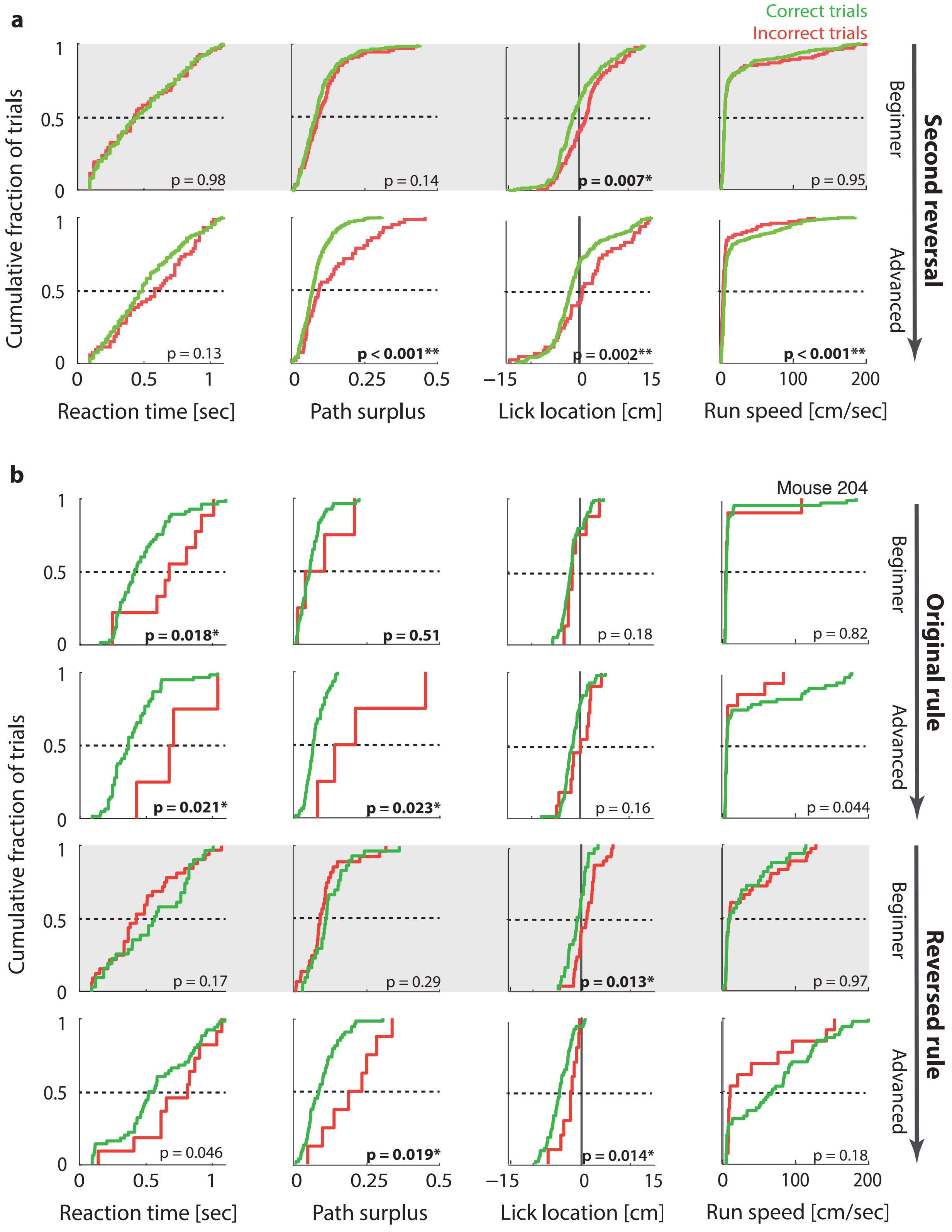

a
Correct trials
Incorrect trials
Cumulative fraction of trials
p = 0.98
p = 0.14
p = 0.007*
p = 0.95
p = 0.13
p < 0.001**
p = 0.002**
p < 0.001**
Beginner
Advanced
Second reversal
Reaction time [sec]
Path surplus
Lick location [cm]
Run speed [cm/sec]
b
Mouse 204
p = 0.018*
p = 0.51
p = 0.18
p = 0.82
p = 0.021*
p = 0.023*
p = 0.16
p = 0.044
p = 0.17
p = 0.29
p = 0.013*
p = 0.97
p = 0.046
p = 0.019*
p = 0.014*
p = 0.18
Original rule
Reversed rule
Beginner
Advanced
Reaction time [sec]
Path surplus
Lick location [cm]
Run speed [cm/sec]

Figure S7: Rule prediction precedes rule execution in task learning and reversal

a) Running average of hit index, PR score and EP index (same as in Fig. 5b) for two additional example animals throughout the second reversal (left) and the original task training (right), respectively. In both cases, the peak of the error prediction index again preceded a drastic increase in task performance.

b) Height of the first peak in EP index that is used to determine the onset of correct rule anticipation (see a, as well as Figs. 5 and 6), computed for the original task learning and two rule reversals. Dots: Measured EP index peak height. Black lines connect measurements for the same animal. Red crosses: Average across animals. Note that the height of the EP index after rule reversals does not appear to be smaller than that in the original task.

c) Distribution of the ratios between the EP index peak height in the first or second rule reversal, and the EP index peak height in the original task. Red arrow: Average of all 10 ratios (5 animals x 2 reversals). Note that the EP index peak after rule reversals tends to be equal to, or even slightly higher, than that in the original task, as indicated by a ratio close to, or larger than, 1. This suggests that after a rule reversal, animals did not only merely abandon the previous task rule, which would lead to an EP index close to zero. Instead, they appeared to actively adopt (and therefore anticipate) the new rule, resulting in a large positive peak of the EP index.

Figure S7

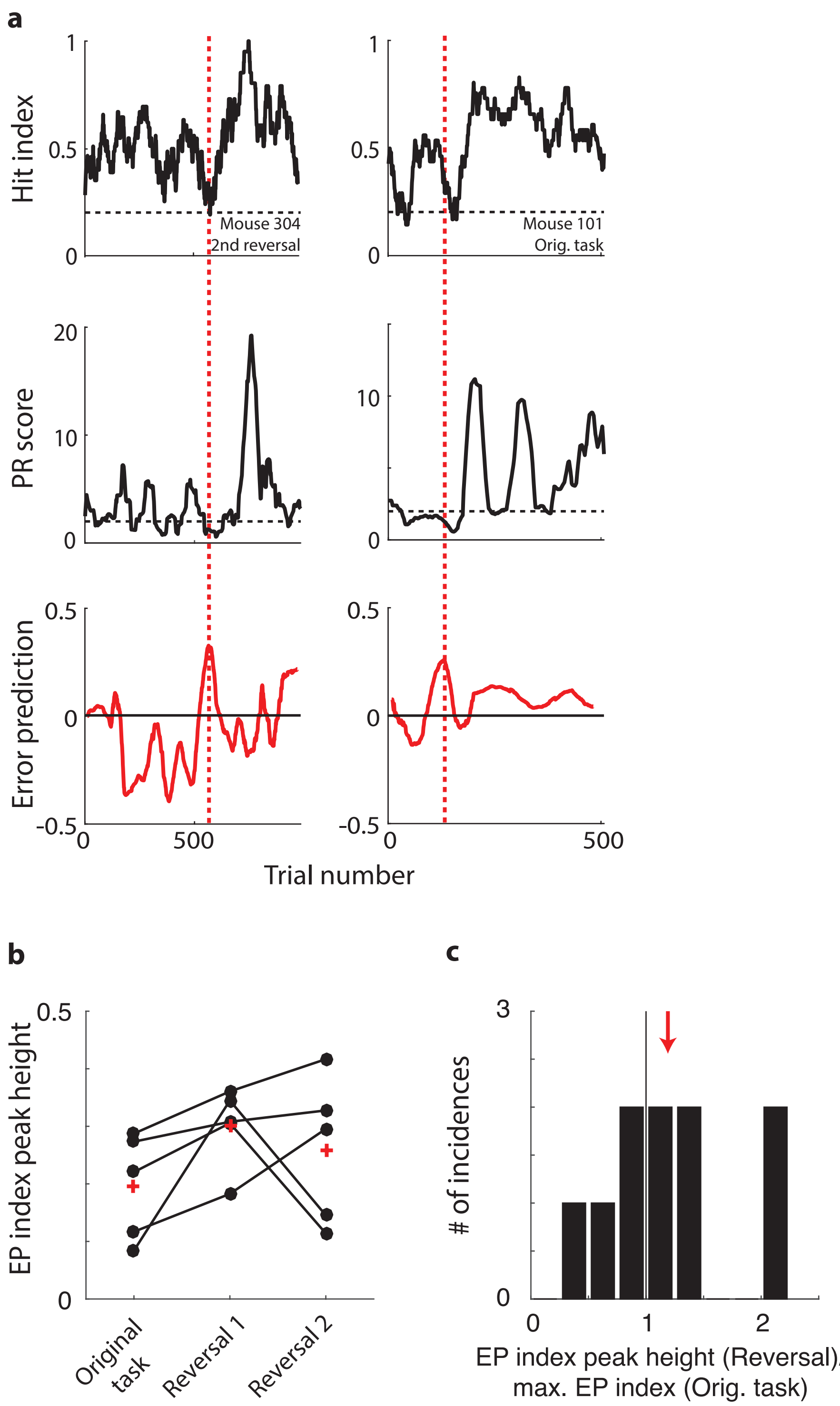
a
Hit index
1
0.5
0
Mouse 304
2nd reversal
Mouse 101
Orig. task
PR score
20
10
0
Error prediction
0.5
0
-0.5
500
Trial number
b
EP index peak height
Original task
Reversal 1
Reversal 2
c
# of incidences
3
0
1
2
EP index peak height (Reversal)/
max. EP index (Orig. task)

Figure S8: Animals make more inadvertent errors during High-Alert phases

a) Example distributions of the PR score across original task (left-hand panels), first rule reversal (center panels) and second rule reversal (right-hand panels) for two example mice. Red arrows mark the boundary between the two peaks of the bimodal distribution (for details on testing the bimodality of the distributions and determining the boundary between peaks, see Methods).

b) Three examples of the relation between PR score and EP index over the course of training. Multicoloured trajectory: PR score and EP index (both smoothed with a 15-trial averaging window) throughout one reversal training. Warmer colours denote trials later in the training process. Vertical dashed line: Cut-off between Low-Alert and High-Alert states (based on the bimodal distribution of PR scores; see Methods). Horizontal dashed line: Zero-point of the EP index, denoting the cut-off between anticipated and inadvertent errors. Note that Low-Alert phases (i.e. parts of the trajectory that fall left of the dashed vertical line) can be associated with a broad range of different EP indices, i.e. with both anticipated and inadvertent errors. In contrast, High-Alert phases (marked by the trajectory moving sharply to the right) generally go along with a negative or low EP index, indicating that the errors that occur during this time are largely inadvertent. Black arrows point out instances of such High-Alert phases.

c) Relation between the proportion of anticipated errors made during spontaneous states of high and low alertness (same as Fig. 7b), but with the initial trials of task learning progressively removed. Shown are five animals in the original task (black dots) and two reversals (dark and light grey dots, respectively), with three data points missing due to an insufficient overall number ($n < 5$) of error trials. Left panel: Proportion of anticipated errors computed based on all trials beginning at training stage 5 (as in Fig. 7b), but with

the first 50 trials not taken into account. Middle panel: Same, but with the first 75 trials beginning at training stage 5 removed. Right panel: Same, but with the first 100 trials removed. In all three cases, data points are concentrated above the diagonal (*$p < 0.05$; based on t-test for dependent samples). This indicates that the proportion of anticipated errors is significantly smaller for high than low alertness trials, whether animals were still somewhat learning the task (Fig. 7b) or had already had intermediate (left panel) to extensive (right panel) practice.

Figure S8

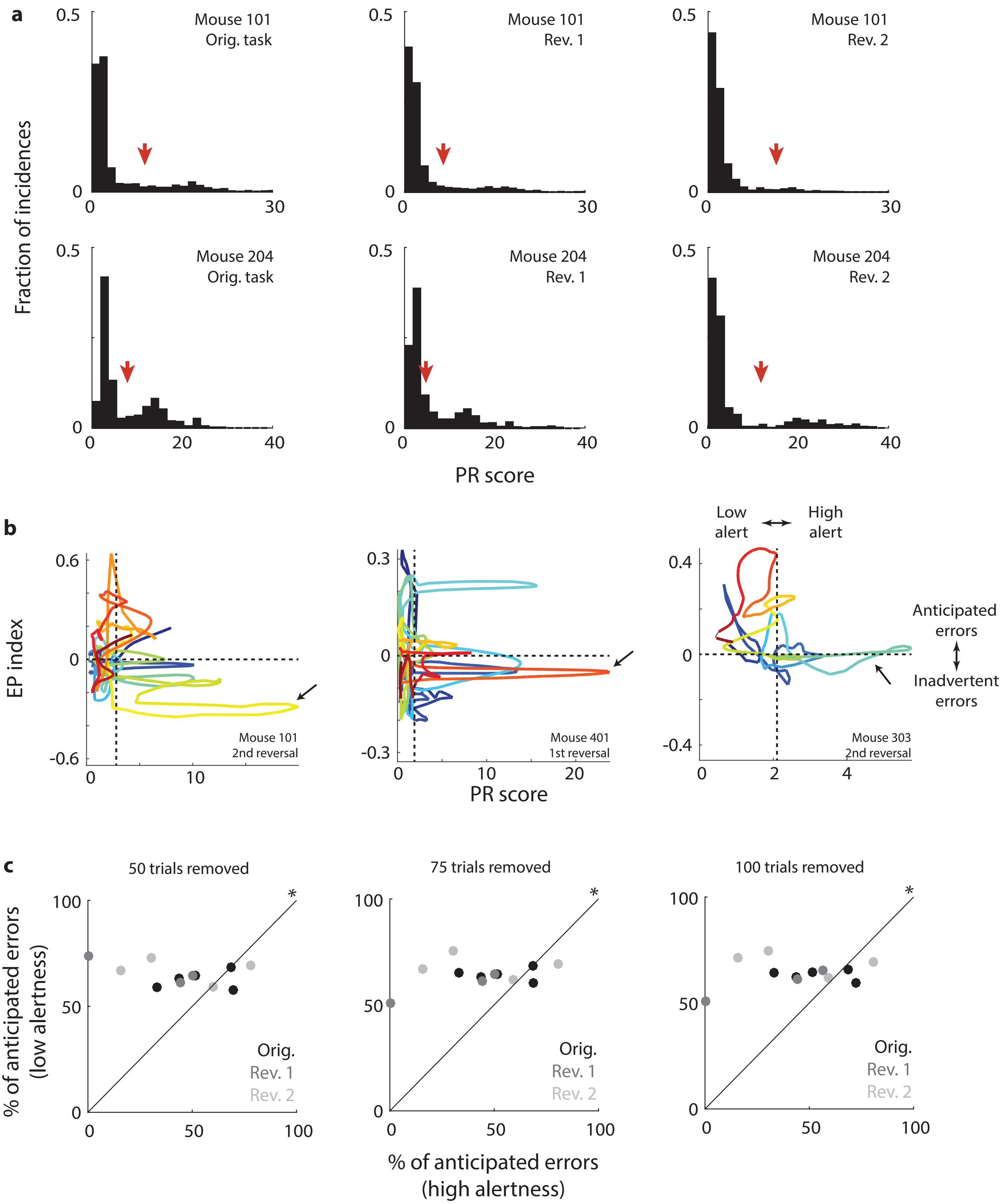
a
Mouse 101
Orig. task
Mouse 101
Rev. 1
Mouse 101
Rev. 2
Mouse 204
Orig. task
Mouse 204
Rev. 1
Mouse 204
Rev. 2
Fraction of incidences
PR score
b
Low alert
High alert
Anticipated errors
Inadvertent errors
EP index
Mouse 101
2nd reversal
Mouse 401
1st reversal
Mouse 303
2nd reversal
PR score
c
50 trials removed
75 trials removed
100 trials removed
Orig.
Rev. 1
Rev. 2
% of anticipated errors (low alertness)
% of anticipated errors (high alertness)

Supplementary Movie S1 – Example of training progression

Sequence of clips showing one animal (Mouse 302) going through all training stages within four sessions. Clips were excerpted from full-session recordings for session 1 and 2 (recorded on March 7 and 9, 2016, respectively) and represent the early stages of each training step. The final clip was recorded during session 4 (on March 11, 2016) with a camera phone to show the task more fully than with a static webcam.
